# *IPD3*, a master regulator of arbuscular mycorrhizal symbiosis, affects genes for immunity and metabolism of non-host *Arabidopsis* when restored long after its evolutionary loss

**DOI:** 10.1007/s11103-024-01422-3

**Published:** 2024-02-18

**Authors:** Eli D. Hornstein, Melodi Charles, Megan Franklin, Brianne Edwards, Simina Vintila, Manuel Kleiner, Heike Sederoff

**Affiliations:** Department of Plant and Microbial Biology, North Carolina State University, Raleigh, NC 27695 USA

**Keywords:** Mycorrhizae, Symbiosis, Non-mycorrhizal, Genetic engineering, Systems biology, Plant transcriptomics

## Abstract

Arbuscular mycorrhizal symbiosis (AM) is a beneficial trait originating with the first land plants, which has subsequently been lost by species scattered throughout the radiation of plant diversity to the present day, including the model *Arabidopsis thaliana*. To explore if elements of this apparently beneficial trait are still present and could be reactivated we generated *Arabidopsis* plants expressing a constitutively active form of *Interacting Protein of DMI3*, a key transcription factor that enables AM within the Common Symbiosis Pathway, which was lost from *Arabidopsis* along with the AM host trait. We characterize the transcriptomic effect of expressing *IPD3* in *Arabidopsis* with and without exposure to the AM fungus (AMF) *Rhizophagus irregularis*, and compare these results to the AM model *Lotus japonicus* and its *ipd3* knockout mutant *cyclops-4*. Despite its long history as a non-AM species, restoring *IPD3* in the form of its constitutively active DNA-binding domain to *Arabidopsis* altered expression of specific gene networks. Surprisingly, the effect of expressing *IPD3* in *Arabidopsis* and knocking it out in *Lotus* was strongest in plants not exposed to AMF, which is revealed to be due to changes in *IPD3* genotype causing a transcriptional state, which partially mimics AMF exposure in non-inoculated plants. Our results indicate that molecular connections to symbiosis machinery remain in place in this nonAM species, with implications for both basic science and the prospect of engineering this trait for agriculture.

**Supplementary Information:**

The online version contains supplementary material available at 10.1007/s11103-024-01422-3.

## Key message

*Arabidopsis* lost its ability to host mycorrhizal symbiosis over 65 million years ago. Restoring expression of an important mycorrhizae-related gene from a legume into *Arabidopsis* regulated genes for defense and stress.

**Supplementary Information:**

The online version contains supplementary material available at 10.1007/s11103-024-01422-3.

## Introduction

Arbuscular mycorrhizae (AM) are formed during symbiosis between host plants and soil fungi. In AM, the plant provides carbon in the form of lipids and sugar to the fungus, and receives water and nutrients in return (Oldroyd 2013; Rich et al. 2017). AM can also confer resistance to pathogens and abiotic stress (Ceballos et al. 2013; Aliyu et al. 2019; Begum et al. 2019; Ramírez-Flores et al. 2020). AM are conserved in the majority of plant species from a single origin through the present day, and are thought to have aided in the first colonization of land (Wang et al. 2010; Delaux et al. 2013; Genre et al. 2020). However, multiple independent losses of the AM trait and its genetic machinery have occurred in diverse plant clades (Cosme et al. 2018). Why a trait considered to be beneficial has been so often lost is a puzzle important for not only basic understanding of AM symbiosis' role in plant resilience, but also for potential crop improvement through re-engineering the trait (Hornstein and Sederoff 2023).

*Arabidopsis thaliana* is one of the ~ 7% of plant species that lack the ability to form AM and which have not replaced them with an alternative endosymbiosis (Veiga et al. 2013; Brundrett 2017; Brundrett and Tedersoo 2018; Cosme et al. 2018; Radhakrishnan et al. 2020). Other such non-mycorrhizal (nonAM) plants include economically important species in the Brassicaceae and Amaranthaceae (Brundrett 2017). Proposed reasons for AM loss include changes in root morphology and lifestyle, altered insect and pathogen resistance, and the carbon cost of supporting the symbiont (Sikes 2010; Field et al. 2012; Brundrett 2017; Ma et al. 2018; Poveda et al. 2019). Little experimental validation exists for drivers of AM loss in individual species, let alone an overarching explanation for which species lose AM, why, and when. The proximate genetic causes for mycorrhizal loss, however, are quite clear: a shared subset of specific genes is deleted in all independent nonAM evolutions (Delaux et al. 2014; Radhakrishnan et al. 2020).

Gene losses in nonAM plants include the Common Symbiosis Pathway (CSP) that mediates signal perception and transduction (Cope et al. 2019; Radhakrishnan et al. 2020; Genre et al. 2020). Member genes lost in *Arabidopsis* include the cell-surface receptor *SymRK*, the ion channel *DMI1*, the kinase *DMI3*, and the transcription factor *IPD3* (Delaux et al. 2014; Bravo et al. 2016; Radhakrishnan et al. 2020). These genes mediate a signal transduction pathway leading from AMF perception to activation of DMI3 by calcium (Demchenko et al. 2004; Pan et al. 2018; Feng et al. 2019). DMI3 then phosphorylates IPD3, enabling its DNA-binding activity for regulation of downstream targets (Oldroyd 2013; Pimprikar and Gutjahr 2018). Outside the CSP, groups of genes involved in lipid flux from plant to AMF and in vesicle trafficking to the arbuscule are also lost (Radhakrishnan et al. 2020).

*IPD3 *(*Interacting Protein of DMI3*) is notable among lost genes in nonAM plants. While most such genes belong to families with non-AM-specific homologs, *IPD3* does not, suggesting its function is distinct to symbiosis. Genetic work has also demonstrated the powerful role of this gene in turning CSP signaling into regulation of AM response genes. The *ipd3* knockout phenotype is near-complete elimination of mycorrhization (Watts-Williams and Cavagnaro 2015). Ectopic expression of IPD3 constitutively activated via phosphomimicking (*IPD3*^*S50D*^) or truncation to the DNA-binding domain (*IPD3*^*Min*^), however, results in symbiosis-like gene regulation even in the absence of a microbial signal or upstream CSP genes (Singh et al. 2014; Pimprikar et al. 2016).

Here, we express *IPD3* in *Arabidopsis* based on knowledge of its uniquely powerful function in AM. By restoring expression of this gene that was present in the mycorrhizal ancestors of *Arabidopsis* before loss of AM in the Brassicaceae, we apply a novel means of identifying retained or unrecognized connections between AM and other genetic networks conserved in nonAM plants. Characterization of *IPD3*-expressing *Arabidopsis* targets two specific questions. First, does *IPD3* have roles outside of its canonically narrow function in AM establishment? Second, does *IPD3* retain functional molecular relationships when restored to *Arabidopsis*, which lost its ancestral copy of this gene along with the trait approximately 65 million years ago (Hohmann et al. 2015)? If so, could reconnecting such relationships alter the response to AMF or even restore symbiosis?

**Fig. 1:**
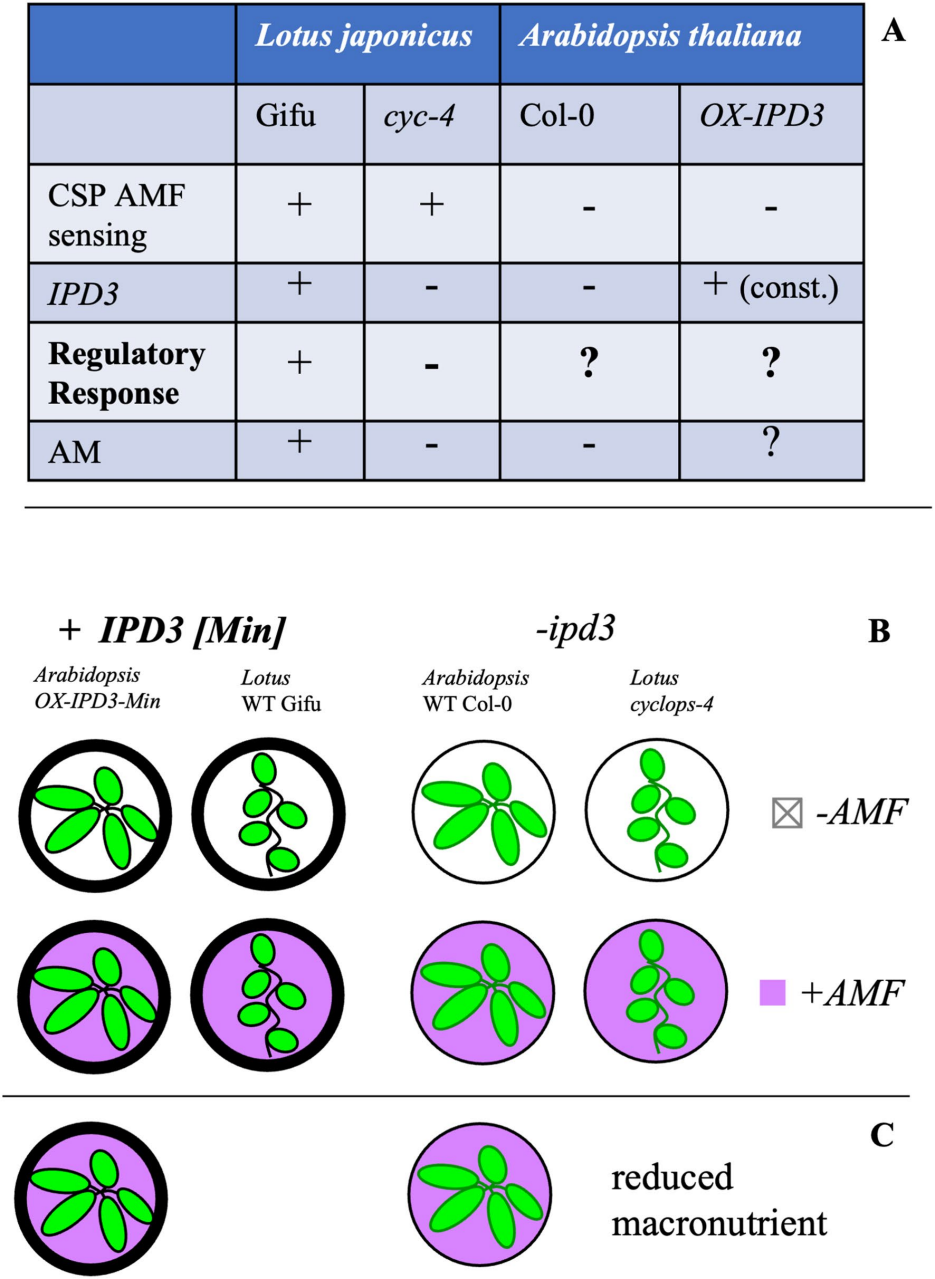
Experimental design comparing different species and genotypes. **A** The AM-host *Lotus japonicus* wildtype (ecotype Gifu) and *IPD3* knockout (*cyclops-4; cyc4*) genotypes differ in their ability to establish AM. *Arabidopsis thaliana* as a non-host species was engineered to express the DNA binding domain of IPD3 (*OX-IPD3*^*Min*^). **B** Phenotypic and transcriptomic analysis was carried out on the genotypes with or without AM inoculation and **C** under high and low macronutrient-containing media. Two independent transgenic lines of *Arabidopsis* were used for analysis in **B** and 1 transgenic line of *Arabidopsis* was used for analysis in **C**

We use transcriptomics to understand the gene-regulatory impact of restoring *IPD3* expression to *Arabidopsis*, and we also compare the inverse case of *IPD3* knockout in a mycorrhizal host plant, via the *cyclops-4* mutant of *Lotus japonicus.* While Lotus has the upstream CSP that normally activates IPD3 in the presence of AMF, the *cyclops-4* mutant lacks functioning IPD3 and results in Lotus that fails to form mycorrhizae despite having the upstream CSP elements intact (Yano et al. 2008; Singh et al. 2014; Pimprikar et al. 2016). This Lotus mutant phenotypically mirrors wild type Arabidopsis in lacking AM, though Arabidopsis further lacks many other genes as described above (Delaux et al. 2013). We subjected both genotypes of both species to AMF treatment, thus, our experiment generates a factorial set of transcriptome data for every combination of *IPD3* genotype, [non]symbiotic species background, and AMF exposure (Fig. 1). Because AMF cannot colonize or survive in long-term monoculture with nonAM plants, we use a pre-germinated spore treatment as described by Fernández et al. to study the early phase of plant-AMF interaction after 48 h of co-culture (Fernández et al. 2019). This corresponds to the pre-symbiotic phase of AM host plant-AMF interaction where significant CSP activity takes place but little colonization is established (Gutjahr and Parniske 2013).

We additionally examined a set of AMF-exposed *Arabidopsis* plants under low-macronutrient conditions (N, P, K) as a known regulator of symbiotic interactions in host plants (Bonneau et al. 2013; Nouri et al. 2014; Carbonnel and Gutjahr 2014). We perform correlation network and differential expression analyses to identify patterns in transcriptomic data from this experiment, and explore their functional implications using gene ontology (GO).

## Materials and methods

Please see supplemental experimental procedures in Online Resource 1 for additional detail on all sections.

### Generation of transgenic *Arabidopsis*

The coding sequences of MtIPD3, S50D-IPD3, and IPD3-Min (Genbank EF569224.1; Yano et al. 2008; Singh et al. 2014) were synthesized (Integrated DNA Technologies, Research Triangle Park, NC) and assembled by Hi-Fi assembly (New England Biolabs, Ipswich, MA) into a modified pCAMBIA0380 expression construct (Genbank AF234290.1) under control of the Arabidopsis Ubiquitin 10 promoter (Ivanov and Harrison 2014). *35S:mCherry* amplified from pC-GW-mCherry (Genbank KP826771.1) (Dalal et al. 2015) was the selection marker. Arabidopsis were transformed as described by Davis et al. (2009). Seeds were screened by fluorescence and PCR during segregation and lines were brought to homozygosity.

### Protein analysis

Protein was extracted from roots and shoots of 6-week-old seedlings grown on 1/2MS plates. For untargeted shotgun proteomics, 5 samples per line and tissue type were ground and lysed in SDT buffer, then prepared as described in Wiśniewski et al. (2009) before 1D-LC-MS/MS using an UltiMate^TM^ 3000 RSLCnano Liquid Chromatograph and Orbitrap Eclipse Tribrid mass spectrometer (Thermo Fisher) as described in Mordant and Kleiner (2021). Mass spectra were searched against a database of Col-0 *A. thaliana* proteins (Uniprot:UP000006548) using the SEQUEST HT algorithm in Proteome Discoverer. Protein abundance was quantified as normalized spectral abundance factor (NSAF) in Microsoft Excel (Zybailov et al. 2006).

For analysis of specific protein size ranges corresponding to bands observed on blots or predicted protein length, we used in-gel digestion of excised SDS-PAGE prior to LC-MS/MS analysis (GeLC-MS/MS). Only 1 sample per construct was used for GeLC-MS/MS. 30–40 µg of total protein in lysate generated for untargeted proteomics was denatured by heating with Laemmli buffer, then run on an SDS-PAGE 12% separating gel with 5% stacking gel. Excised bands were processed according to Shevchenko et al. (2006), and 10 μL of peptide mixture was injected to LC as described above, connected to an Orbitrap Exploris 480 mass spectrometer (Thermo-Fisher) for MS/MS with the same settings used for untargeted proteomics.

For Western blotting, frozen tissue was hand-ground with extraction buffer, centrifuged, and the supernatant denatured by heating in LDS sample buffer. Protein was run on a 12% Tris-Glycine acrylamide gel, then transferred to PVDF membranes and blocked in TBS+2% BSA with 4 μL/mL Tween-20, then incubated with 1:1000 dilution of custom rabbit anti-IPD3 polyclonal antibody (Genscript, China) followed by 1:2500 donkey anti-rabbit AlexaFluor 488-conjugated secondary antibodies (Thermo Fisher, Waltham, MA) and imaged on a GelDoc SR (Bio-Rad, Hercules, CA).

### Growth phenotyping

Plants were grown in the NCSU phytotron under long day conditions in 8 oz pots filled with SunGro propagation mix (Sungro, Agawam, MA). Pots were hand-watered with deionized water. Seeds were harvested from dried mature plants and hand-cleaned before weighing. The seed yield experiment was repeated 3 times with n = 9–15. The flowering time experiment was performed once with n = 14 for line 312 and n = 15 for all other lines, with plants censused daily for onset of bolting.

### Transcriptome experiment

Seeds were sterilized and grown for 6 weeks under long-day conditions (16 h light/8 h dark, 21/18 ℃) on sterile petri dishes containing either 1/2MS or low-nutrient MS media (Phytotech Labs, Lenexa, KS). Following the protocols established by Fernández et al. (2019) and Mukherjee and Ané (2011), sterile spores of *R. irregularis* (Premier Tech, Canada) were pre-germinated for 1 week at 26 ℃, and a suspension of ~ 200 germinated spores was added to the roots of each AMF-treated plant 48 hours prior to sample collection. Control plants were mock-inoculated with sterile water. Each replicate consisted of the pooled roots of 5 seedlings from the same plate.

RNA was extracted with the Purelink RNA Mini kit and treated with Turbo DNAse (Invitrogen, Waltham, MA), then sequenced by DNBSEQ at BGI Group (China) using strand-specific, poly-A enrichment to obtain 100 bp paired-end reads. *Lotus* and *Arabidopsis* alignments were performed using BBSplit, a multi-reference aligner, to separate plant and fungal reads (Bushnell 2014). Reference genomes used were Araport 11 for *Arabidopsis* (TAIR), the Joint Genomics Institute assembly for *R. irregularis* (Genbank: GCA_000439145.3), the Gifu V1.2 assembly for *Lotus* (GCA_012489685.2), the *IPD3-Min* transformation construct, and the *LjCYCLOPS* sequence. Reads were counted at the transcript level using featureCounts (Liao et al. 2014).

Gene expression networks were constructed using WGCNA v1.69 (Langfelder and Horvath 2008, 2012) with soft-threshold power of 16 (*Arabidopsis*) or 24 (*Lotus*). Correlation coefficients were calculated between the eigengenes of each module and treatment variables to identify significant module-trait relationships, with p < 0.05. Gene Ontology enrichment and hierarchical term clustering was performed with PANTHER (Mi et al. 2013). Terms were filtered by specificity and significance, then subjected to semantic similarity clustering in reviGO (Supek et al. 2011).

Differential expression analysis was performed in R using the edgeR package (Liao et al. 2014; Robinson et al. 2010). The estimateGLMCommonDisp function with FDR-adjusted p-value < 0.05 was used to test for differential expression. Cross-comparison of gene lists was done in Excel.

## Results

### Generation of transgenic *Arabidopsis*

The transcription factor IPD3 consists of an activation domain that can be phosphorylated on Serine-50 upon AM inoculation and a DNA-binding domain essential for transcriptional regulation of downstream effectors (Singh et al. 2014; Jin et al. 2018). To ensure that we can identify transcriptional regulation via IPD3 in the non-host that is likely lacking signaling and activation of IPD3, we generated 7 homozygous transgenic Arabidopsis lines expressing Medicago truncatula *IPD3* (*IPD3*^*Mt*^), 4 lines of phosphomimic *IPD3* (*IPD3*^*S50D*^), and 5 lines expressing *IPD3*^*Min*^ (aa 254-513 of IPD3^Mt^) (Singh et al. 2014). All transgenes were expressed under the *Arabidopsis UBIQUITIN10* promoter (Grefen et al. 2010; Ivanov and Harrison 2014). RT-PCR confirmed RNA expression of all versions of the transgene.

Western blot of root and shoot tissue of T3 individuals confirmed presence of IPD3^Min^ protein, but not IPD3^Mt^ and IPD3^S50D^ (Online Resource 2). We noted that Western blot visualization was difficult to obtain, requiring the precise method described in Online Resource 2, and that root and shoot IPD3^Min^ protein bands appear to run at slightly different size, which might represent influence of unknown post-translational modification or cellular localization that interferes with expression and running conditions. We therefore performed targeted protein sequencing using mass spectrometry to confirm that the protein bands observed on the blot were in fact IPD3^Min^. Excised SDS-PAGE gel bands at the expected size of IPD3^Min^ in shoot and root samples were used for shotgun proteomics (Online Resources 1 and 3). *IPD3*^*Mt*^ and *IPD3*^*S50D*^ lines were analyzed in the same manner, however, and as with Western blot of these lines, protein expression was again not detected. To further confirm that our ability to detect and identify all forms of IPD3 was not affected by gel running conditions or extraction, we then performed untargeted shotgun proteomics of shoots and roots (n = 5) using independent processing methods (Online Resource 1). In untargeted proteomic analysis, IPD3^Mt^ and IPD3^S50D^ were again not detected, while IPD3^Min^ was highly abundant (top 10% NSAF) in respective lines (Online Resource 3). Consequently, we focused only on characterizing the 5 *IPD3*^*Min*^ lines (numbered 303; 308; 310; 312; 357).

In *IPD3*^*Min*^ lines used we confirmed that protein and RNA of the gene of interest were highly expressed relative to native sequences (Online Resources 3 and 15), however, these quantitative data do not provide a direct measurement of transcription factor activity of the protein product.

### Phenotypic effect of *IPD3*^*Min*^ expression in *Arabidopsis*

**Fig. 2:**
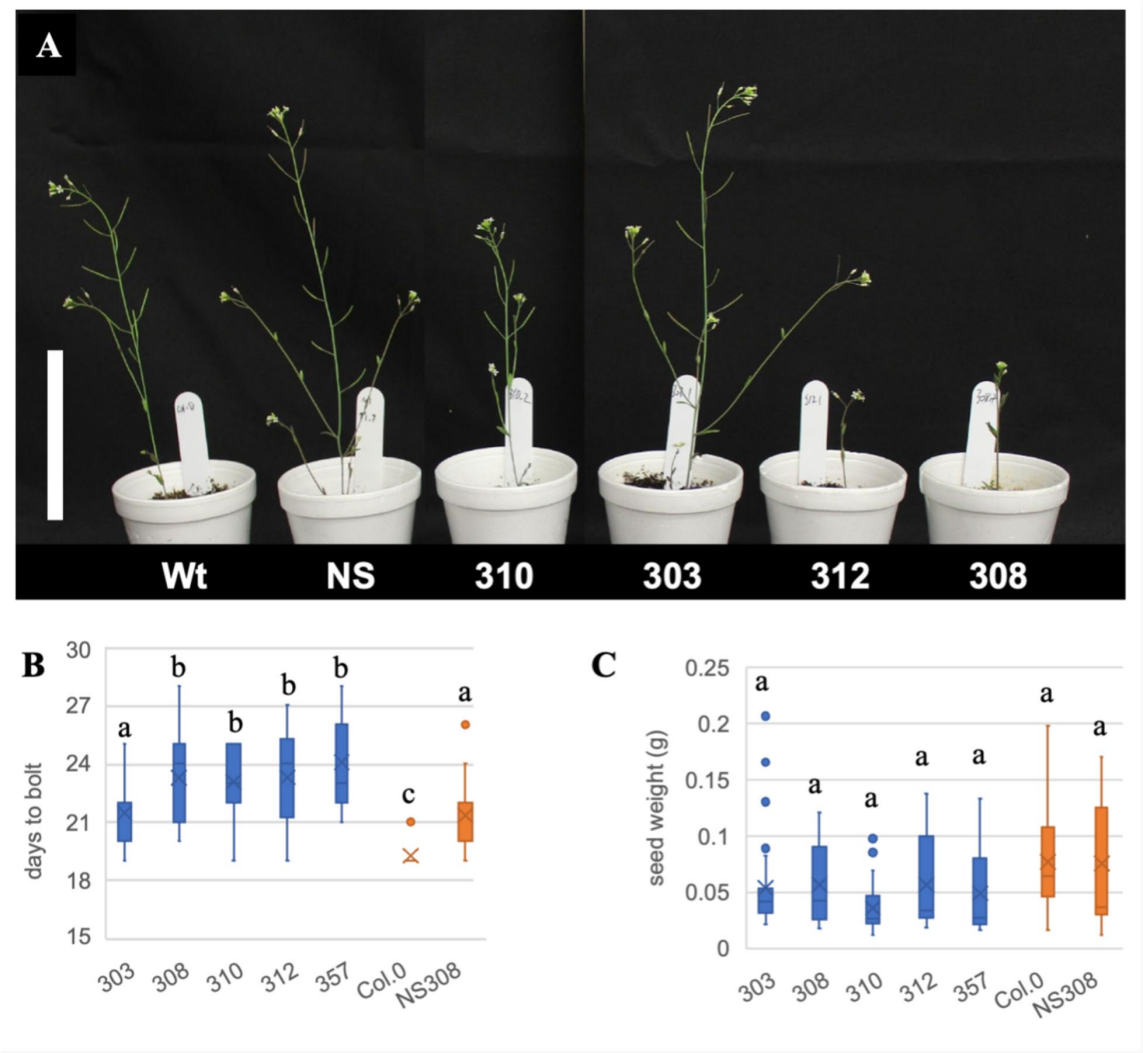
Phenotypes of independent homozygous transgenic lines expressing the DNA-binding domain of IPD3 (IPD3^Min^). **A** Shoots of most *IPD3*^*Min*^ transgenic lines appear shorter, less branched and delayed in development; scale bar 10 cm. **B** *IPD3*^*Min*^ Transgenic lines (blue) had a delayed transition to reproductive development (onset of bolting) relative to nontransgenic controls (orange) (ANOVA, p < 0.05, n = 15), but **C** showed no significant differences in total seed yield (MCM p < 0.05) (in **B** and **C** mean is marked with X and median marked with –, outliers shown as dots) (see Online Resources 4–6)

Plants were surveyed for differences in growth under long-day conditions (Fig. 2, Online Resource 4). All 5 transgenic *IPD3*^*Min*^ lines were significantly slower to initiate the transition to flowering than wild type Col-0, measured as days to onset of bolting (Fig. 2A, B, Online Resource 5). Four of 5 lines (excluding 303) were also significantly slower to flower than a null segregant control (Fig. 2B). Despite flowering differences, no significant difference in seed yield was detected over 3 repetitions of the experiment (Fig. 2C). No difference in germination timing was observed (Online Resource 5). An unexpected strong phenotype was the pink coloration of four of the five *IPD3*^*Min*^ transgenic plant roots, while this color in *IPD3*^*Mt*^ and *IPD3*^*S50D*^ lines was very weak in 4 out of the 11 plants, and visually absent in the remaining 7 (Online Resource 6). In addition, 7 of the 8 independent T1 empty vector lines showed a range of pink coloration in roots. Because anthocyanins or carotenoids can cause this coloration in plant tissues, we performed extraction protocols for those secondary metabolites, but were not able to confirm any measurable quantities. The most likely conclusion is that the color could be caused by the marker mCherry that was used as a reporter gene. This reporter gene was present in the *IPD3*^*Mt*^ and *IPD3*^*S50D*^ constructs as well, where little to no pink coloration was observed, which may relate to the lack of expression and potential silencing of those transgenes as assessed through proteomics (Online Resources 2, 3).

We also monitored *Lotus japonicus* plants used in cross-species comparison for overt differences between wild type (Gifu ecotype) and the *cyclops-4* knockout mutant of *IPD3*. Consistent with prior literature we noted no obvious growth phenotype in the *ipd3* mutant (Yano et al. 2008; Horváth et al. 2011) (Online Resource 7). The genotype of *cyclops-4 Lotus* was confirmed by Sanger sequencing of the *CYCLOPS* genomic region.

### Transcriptional consequences of manipulating *IPD3*

Before a functional AM host trait could be engineered into *Arabidopsis* as a reference plant for Brassicaceae (Hornstein and Sederoff 2023), the potential for evolutionarily conserved interactions and pathways can be explored via comparative transcriptome analysis of non-host and host plants with and without IPD3/IPD3^Min^. We sequenced total mRNA of 6-week-old roots of transgenic *IPD3*^*Min*^, mutant *cyclops-4*, and wild type *Arabidopsis* Col-0 & *L. japonicus* Gifu plants with or without 48 hours of AMF germinated spore treatment prior to collection (Fig. 1). Two independent transgenic lines of *Arabidopsis* (308 and 310) with similar phenotype (Fig. 2, Online resources 4, 6) and *IPD3*^*Min*^ transcript abundance were included in the experiment to account for possible variation due to genomic insertion site effects and epigenetic consequences in transgenic lines (Schnell et al. 2015).

We used statistical methods like Principle Component Analysis (PCA) and Differential Gene Expression as well as machine learning for network analysis to identify genes, pathways and networks in Arabidopsis and Lotus plants that are responsive to AMF inoculation and dependent or independent of IPD3 regulation. The comparison of the species and genotype specific transcriptome analyses provides an insight into those pathways that are still conserved in Arabidopsis after the evolutionary loss of the AM host trait through IPD3 loss. Correlation network construction used quantitative expression of *IPD3*^*Min*^ in each sample of both lines as the primary transgenic trait. In differential expression analysis an *IPD3*^*Min*^ category including only genes independently attested as DEGs in both transgenic lines was used. We also compared Col-0 *Arabidopsis* to a single *IPD3*^*Min*^ transgenic line (308) with AMF inoculation on low-nutrient (LN) MS medium containing 0.5% P, 1% N, and 1% K relative to 1/2 MS (Fig. 1).

In principal component analysis (PCA) of *Lotus*, Gifu transcriptomes are clustered according to AMF treatment along PC1 (Online Resource 8). *cyclops-4* transcriptomes were separated from those of Gifu plants along PC2. Interestingly, both AMF-treated and untreated *cyclops-4* transcriptomes clustered along PC1 with those of AMF-treated Gifu plants. In *Arabidopsis*, *IPD3*^*Min*^ transgenic lines clustered relative to Col-0 along PC1 (Online Resource 8). Line 308 clustered farther from Col-0 than line 310, most likely due to the higher *IPD3*^*Min*^ expression in individuals of this line (Online Resources 8, 9). Col-0 plants clustered by AMF treatment along PC2, but there was no clear separation by treatment among *IPD3*^*Min*^ lines (Online Resource 8). PCA of the low-nutrient experiment also shows strong clustering by genotype (Online Resource 10). In conclusion, the variance in gene expression changes in wild type *Lotus* and *Arabidopsis* plants corresponds more to the inoculation with AMF, while gene expression variation in response to presence or absence of IPD3/IPD3Min is less affected by presence or absence of AMF and this variance is explained by a different component.

### Correlation network and gene ontology analysis

**Fig. 3:**
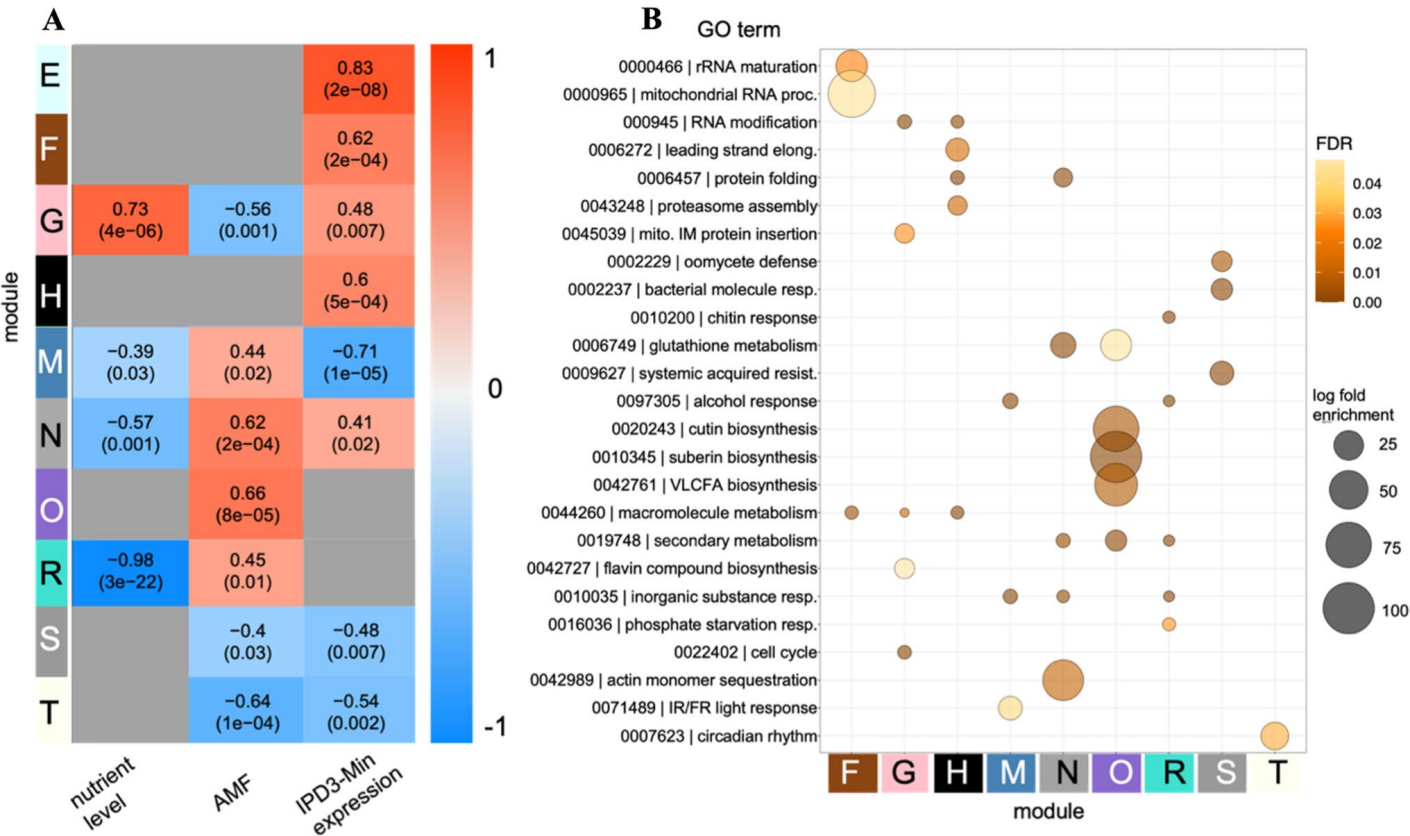
Correlation network modules and gene ontology analysis for transcription of *Arabidopsis thaliana IPD3*^*Min*^ and Col-0 genotypes across AMF and low-nutrient treatments. **A** Selected correlation modules, including all those significantly correlated to *IPD3*^*Min*^ expression. Modules are labeled E–T in order of appearance in the full network (Online Resource 7). Color of cells within the heatmap reflects correlation of that module to trait values; p-value of the module-trait correlation is listed in parentheses. **B** Gene Ontology enrichment of all modules shown in **A**; terms have been reduced by applying a cutoff for the top 3 most-enriched and most-significant terms in each module followed by overlap analysis to establish representative terms. Color intensity corresponds to FDR-corrected p-value of term enrichment and circle area corresponds to scale of enrichment. (See Online Resource 11, Online Resources 12, 14, S10)

**Fig. 4:**
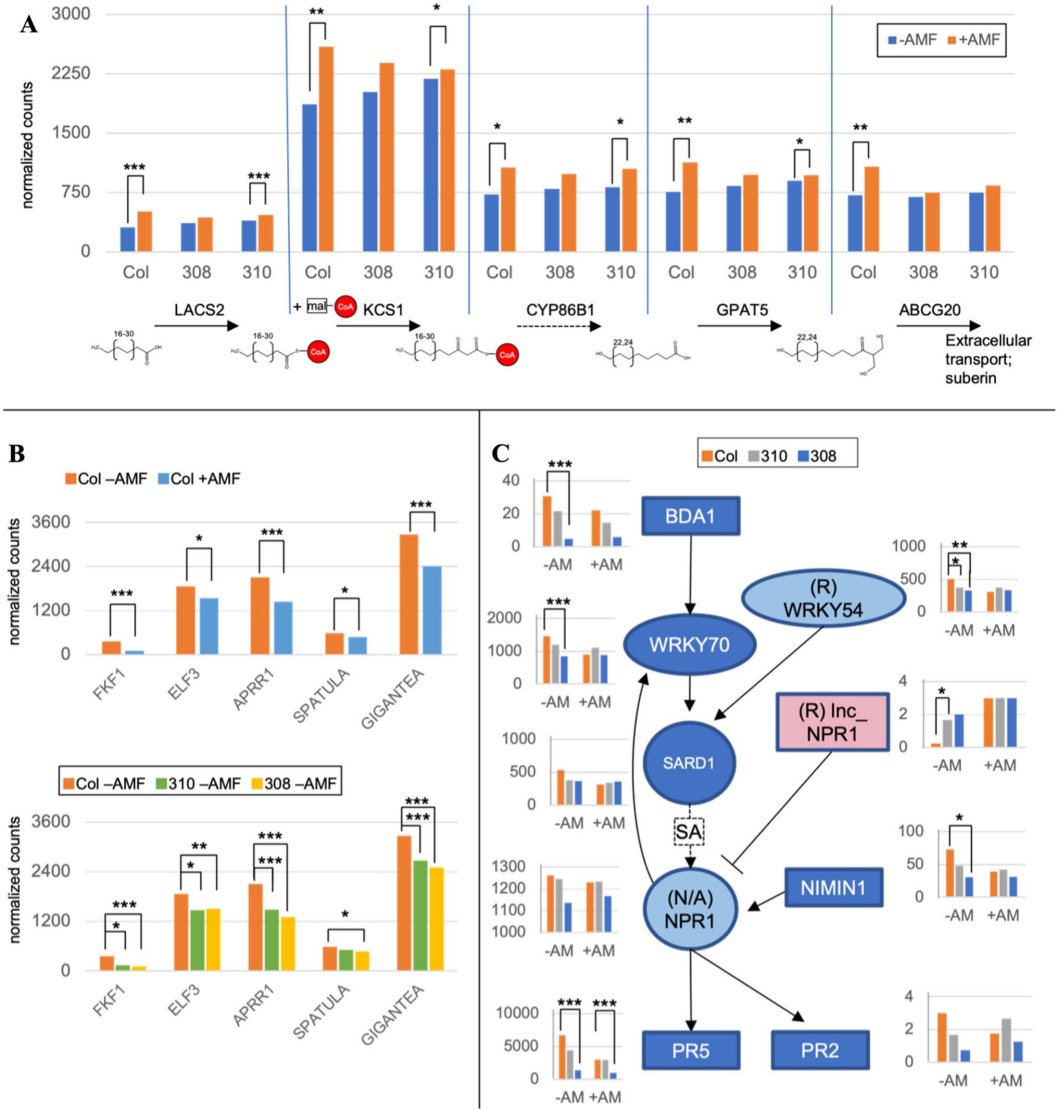
Targeted analysis for pathways of interest within correlation network modules. All genes included in this figure are statistically significant members of the respective network module (having module-trait correlations at p_GS < 0.05 as indicated in Fig. 3) at p_kME < 0.05. Significance reflects 1-tailed t-test of group means for transcript counts of specific genes, a complementary measure which is calculated independently from both the correlation network and untargeted DEG analysis; n = 3–5, *p < 0.05; **p < 0.01; ***p < 0.005. Standard Error of all treatment groups is provided in Online Resource 15 in addition to t-test results. Some interactions in pathway maps have been omitted for clarity. **A** Expression of a subset of individual genes belonging to module O related to extracellular wax synthesis. **B** Expression of well-known circadian clock related genes in module T. **C** Pathway map of the WRKY70-SA-NPR1-mediated defense response with expression of individual genes. Genes belong to module S unless marked otherwise in parentheses. (See Online Resources 12, 15). Gene acronyms are expanded as follows: **A** LACS2, Long-chain Acyl-CoA Synthetase 2; KCS1, 3-Ketoacyl-CoA Synthase 1; CYP86B1, Cytochrome P450, Family 86, Subfamily B, Polypetide 1; GPAT5, Glycerol-3-phosphate sn-2-Acyltransferase 5; ABCG20, ATP-binding Cassette G20. **B** FKF1, Flavin-binding, Kelch Repeat, F Box 1; ELF3, Early Flowering 3; APRR1, Pseudo-response Regulator 1; Spatula; Gigantea. **C** BDA1, Bian Da 1; WRKY70, WRKY DNA-binding Protein 70; WRKY54, WRKY DNA-binding Protein 54; SARD1, SAR Deficient 1; NPR1, Nonexpressor of PR Genes 1; lnc_NPR1, antisense long non-coding RNA of NPR1; NIMIN1, NIM1-interacting 1; PR5, Pathogenesis-related Gene 5; PR2, Pathogenesis-related Gene 2

We used weighted gene co-expression network analysis (WGCNA) as a transcriptome analysis tool which captures global expression patterns reflective of the underlying reality of coordinated expression of many genes affected by multiple factors (Langfelder and Horvath 2008, 2012). WGCNA clusters the transcriptome into modules with similar expression profiles which suggest they may regulate each other or are co-regulated. WGCNA also correlates expression of a representative eigengene for each module to traits and treatments, in this case quantitative *IPD3*^*Min*^ expression, AMF treatment, and nutrient level. The two-step process of module construction and module-trait correlation makes WGCNA sensitive to links between potentially subtle expression changes at the gene level and experimental variables, and can be used to guide more targeted analysis. WGCNA analysis incorporates transgene expression agnostic of categorical information about the 2 transgenic lines, instead reflecting how well expression of native Arabidopsis genes correlate to IPD3^Min^ across a range of variation in transgene expression levels in each individual plant. WGCNA of *Arabidopsis* identified 22 co-regulatory modules (Online Resources 11, 12). Figure 3A includes all modules significantly correlated to *IPD3*^*Min*^ expression, and selected modules correlated to AMF treatment and nutrient level. We used gene ontology (GO) enrichment to link module membership to biological functions (Fig. 3B). We also performed targeted pathway analysis of specific gene members within modules to better connect these broad patterns to physiological functions (Fig. 4). Statistical significance in Fig. 4 is a hypothesis test of whether mean expression of the given gene differs between specific treatment groups, an independently calculated and complementary measure to the network analysis correlations which establish module membership.

The WGCNA analysis identified clusters of co-regulated transcripts that were either responsive to the nutrient level, the exposure to AMF, the expression of IPD3Min, or combinations of those treatments. Further GOterm analysis of those specific clusters showed the potential functional connections of genes that were differentially co-regulated in response to either treatment of combinations of those. Only one cluster of transcripts (cluster O) significantly correlated only with AMF inoculation in Arabidopsis, and three clusters (E, F, H) significantly correlated only with IPD3Min expression. One cluster (M) showed positive correlation with AMF inoculation but negative correlation with IPD3^Min^ expression suggesting repression of those genes by IPD3^Min^.

### The *Arabidopsis* response to AMF includes altered defense and lipid metabolism

Despite its nonAM status, *Arabidopsis* transcriptome networks showed 10 modules of co-regulated genes with trait correlations to AMF treatment, indicating a clear transcriptional response to the fungus (Online Resource 11). We considered modules O and R that correlate to AMF treatment but not *IPD3*^*Min*^ expression to reflect portions of the native *Arabidopsis* AMF response that are unaffected by *IPD3*^*Min*^ expression. Expression of genes in module R positively correlated with AMF treatment but negatively correlated with nutrient level. Top GO terms in this module R related to both nutrient stress (GO:0016036; phosphate starvation) and biotic interactions (GO:0010200; chitin response) (Fig. 4B, Online Resources 13, 14).

Module O expression correlated solely to AMF treatment, independent of IPD3^Min^ genotype and was highly enriched for terms related to biosynthesis of extracellular lipids (GO:0010143; cutin biosynthesis, GO:0010345; suberin biosynthesis, GO:0042761; very long-chain fatty acid biosynthesis) (Fig. 3B). These genes fall along the biosynthetic pathway of 2-monoacylglycerols (2-MAGs), which affect pathogen and stress resistance as components of cutin and suberin (Fig. 4A, Online Resources 12, 14). 2-MAGs are also the primary form of carbon exported to AMF fungus from AM host plants (Luginbuehl et al. 2017; Rich et al. 2017). Mean expression of all tested genes in the pathway was significantly higher in AMF-treated Col-0 plants than in untreated plants (Fig. 4A). Significantly higher expression was also detected in some but not all AMF-treated transgenic lines for the same genes. This indicates that upregulation of lipid biosynthetic genes in AMF-treated *Arabidopsis* is *IPD3*^*Min*^*-*independent, consistent with the module’s correlation to AMF treatment but not *IPD3*^*Min*^ expression in Fig. 3A. The gene level comparisons of transcript abundance for this module shows that both independent transgenic lines (308 and 310) have similar transcript abundance differences to Col-0 and are comparable in their response prior to AMF treatment (Fig. 4B, C). Other genes in module O also function in lipid synthesis, including *GPAT6*, a functional homolog of *RAM2* which is strictly required for AM in host species (Online Resource 12) (Gobbato et al. 2013; Dai et al. 2022). While it is a member of module O, AMF-mediated increase of GPAT6 gene expression was only significant in the IPD3^Min^ line 308, but not in Col-0 nor in the IPD3^Min^ line 310 (Online Resource 15).

### *IPD3*^*Min*^ expression imitates AMF treatment in Arabidopsis Col-0 plants

Transcription in module T correlated negatively to AMF treatment and *IPD3*^*Min*^ expression (Fig. 3A). This is consistent with *IPD3*^*Min*^ expression enhancing the effect of AMF treatment or replicating the effect of AMF in untreated samples. A similar pattern was present in modules N (both positive) and S (both negative) (Fig. 3A). Module T contains only 56 genes and was enriched solely for terms related to circadian rhythm (GO:0007623; circadian rhythm, GO:0048511; rhythmic process) (Fig. 3B, Online Resource 14). Members of this module include components of the *CONSTANS-FT* daylength sensing system (Online Resources 12, 15) (Takagi et al. 2023). As shown in Fig. 4B, AMF treatment of Col-0 plants results in significantly reduced expression for genes across the pathway, and *IPD3*^*Min*^ expression replicates this effect even in the absence of AMF.

Module S transcription was negatively correlated with both *IPD3*^*Min*^ and AMF treatment, and is enriched for GO terms related to pathogen defense and systemic acquired resistance (SAR) (GO:0002229, oomycete defense; GO:0002237, bacterial molecule response; GO:0009627, systemic acquired resistance) (Fig. 3). This module contains the key defense regulator *WRKY70*, which mediates selection of defense responses by positively regulating salicylic acid (SA) immunity and negatively regulating jasmonic acid (JA) related pathways (Li et al. 2006). As shown in Fig. 4C, genes acting up- and downstream of *WRKY70* in SA-mediated defense are also present in this module, including cell-surface ankyrin protein *BDA1*, and pathogen response gene *PR2* (Thomma et al. 2001; Yang et al. 2012a). *NPR1*, another key positive regulator of SA-mediated defense was detected in the transcriptome but not placed in a network module (Fig. 4C) (Thomma et al. 2001).

Several SA defense genes (*BDA1; WRKY70; WRKY54; NIMIN1; PR5*) were significantly downregulated in one or both transgenic lines even in the absence of biotic interaction with AMF (Fig. 4C). This suggests that *IPD3*^*Min*^ expression in isolation can replicate the suppressive effect of AMF interaction on SA-related defense. Consistent with downregulation of SA-related genes, we also found evidence of antagonistic crosstalk between jasmonic and salicylic acid-mediated defenses (Li et al. 2006; Hou and Tsuda 2022). Module R, which in contrast to Module S was upregulated in response to AMF, is functionally enriched for jasmonic acid (JA) signaling (GO:009753) (Online Resource 14). JA genes upregulated by AMF in module R included upstream (jasmonate methlytransferase *JMT*) and downstream (chitinases *PR3* and *PR4*) members of the JA defense pathway (Samac et al. 1990; Thomma et al. 1998; Seo et al. 2001). Module R also contained a set of genes enriched for salicylic acid signaling, some of which are negative regulators of SA defense such as At1g08667.1 (*lnc_NPR1*), a putative antisense transcript of *NPR1* whose upregulation would correspond to downregulation of *NPR1* and the SA response (Fig. 4C, Online Resource 15). Other SA-related genes were placed into module R due to expression correlation at the individual sample level, but upon comparison of treatment group means matched the pattern of downregulation in module S; this included *WRKY54*, a co-regulator with *WRKY70* of SA synthesis (Chen et al. 2021b) (Fig. 4C, Online Resource 15).

Positive *IPD3*^*Min*^ correlation with modules F, G, H, and N corresponded to GO enrichment for background processes, e.g. GO:0000466, rRNA maturation (Fig. 3). These functions are essential to the organism, but limited in GO analysis from being linked to specific effects. These modules contained 4132 genes, indicating that about 10% of all gene models in the *Arabidopsis* genome were upregulated in connection to *IPD3*^*Min*^ (Online Resource 12). Module E had the strongest correlation to *IPD3*^*Min*^ expression but resulted in no significant GO term enrichment (Fig. 3A, Online Resources 12, 14). Notably, 8 of 25 genes subsequently identified as *IPD3-*responsive via differential transcription analysis in Fig. 6B belonged to module E and are discussed in later sections (Online Resource 16).

### Network analysis of *Lotus* transcriptomes

We also constructed a correlation network for *Lotus*, which is available in Online Resources 17 and 18. A distinctive feature of the *Lotus* network is that while expression of 8 modules correlated with *IPD3* genotypes and 15 modules correlated with the AMF treatment, no modules correlated with both treatments. We confirmed transcription of CSP and AMF marker genes including *CCaMK, NSP2,* and *PT4,* however, all detectable CSP genes were placed into network modules not correlated to either treatment.

### Cross-species comparison of differential transcription

The major question of our experiment was whether elements of the AMF response network are still present in the non-host Arabidopsis after the evolutionary loss of the transcription factor IPD3. To directly characterize transcript abundance changes related to the presence or absence of the respective IPD3/IPD3^Min^ versions in a manner that enables cross-comparison of species, we conducted differential transcript abundance analysis for genotype and AMF treatment in *Lotus* and *Arabidopsis*.

**Fig. 5:**
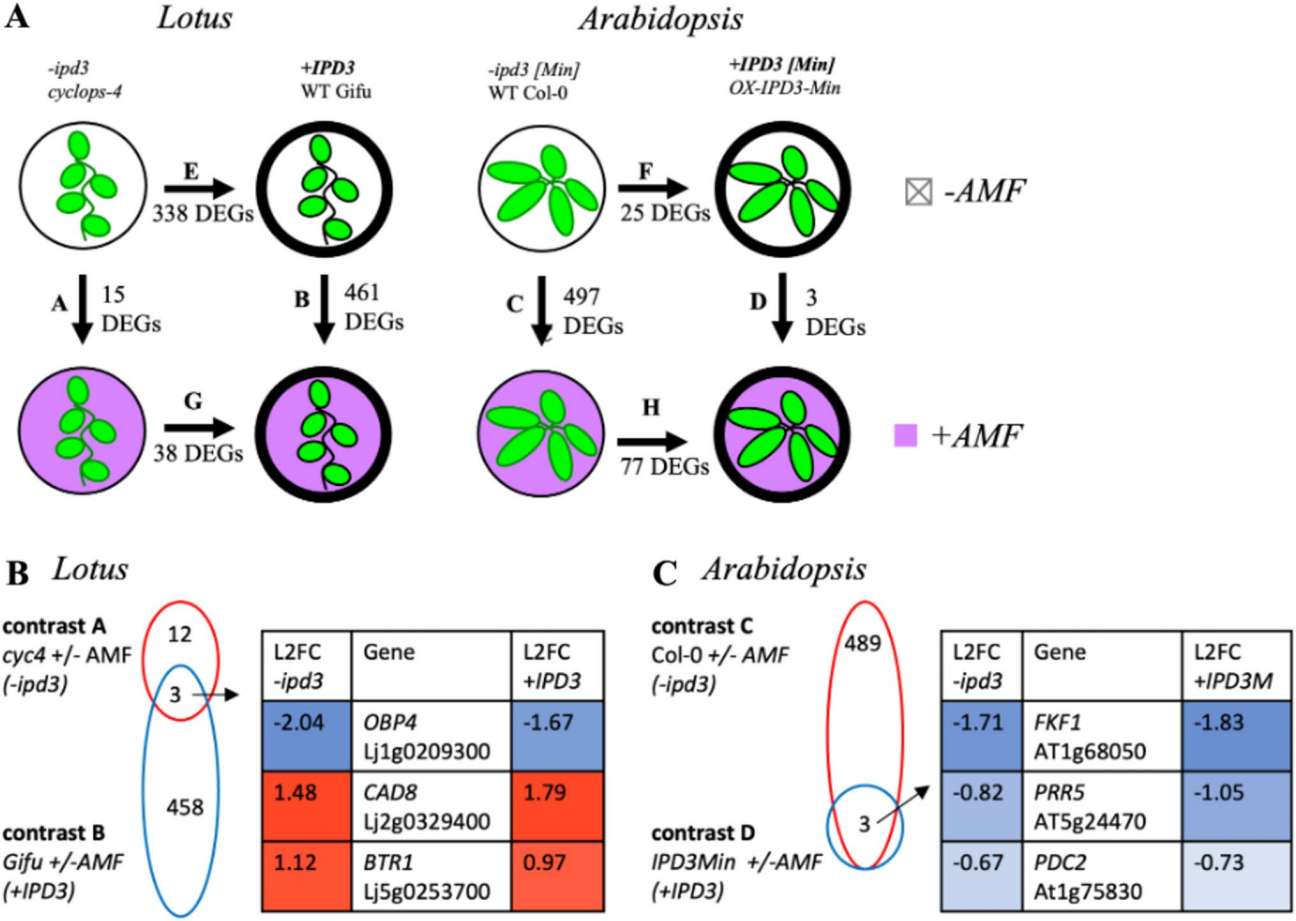
Differential transcription contrasts for *IPD3[Min]* expression with and without AMF treatment in two species. **A** Overview of all contrasts executed and number of DEGs detected. Contrast labels A–H in **B** and **C** and Fig. 6 correspond to those noted in **A**. DEGs reported for the *+IPD3Min* genotype in *Arabidopsis* are those attested in both of the individually conducted contrasts for the 2 transgenic lines 308 and 310. **B** Comparison of DEGs resulting from AMF treatment in *−ipd3* (*cyc4*) and *+IPD3* (Gifu) genotypes of *Lotus.* L2FC is Log_2_(fold change) of mean expression. **C** Comparison of DEGs resulting from AMF treatment in −*ipd3* (Col-0) and *+IPD3* (OX-*IPD3Min*) genotypes of *Arabidopsis.* Differential transcription is expressed as log_2_(fold change) (L2FC); more-positive numbers indicate higher expression and more-negative numbers indicate lower. (See Online Resource 16)

The question to answer in this cross-species comparison was which genes are IPD3-dependent in their response to AMF inoculation in the host plant (Lotus) and then compare it to the non-host Arabidopsis (Fig. 5A, Online Resource #19) with the goal to identify genes that still can be regulated by IPD3^Min^. Transgenic expression used to create the +IPD3^Min^ genotype in Arabidopsis introduces two additional factors which make this genotype only partially parallel to the Lotus wild type. First, the transgene is constitutively expressed under the Ubiquitin promoter and constitutively active at the protein level due to truncation of the activation domain, as opposed to inducible as in Lotus (Pimprikar et al. 2016; Singh et al. 2014). Second, Agrobacterium mediated transformation randomly inserts the transgene into different genomic locations in different independent lines, resulting in differing expression levels and potential effects on native genes (Edwards et al. 2022). To limit influence of such across-line variation, in the primary analysis we considered only those Arabidopsis DEGs independently attested for a given contrast in both transgenic lines (Fig. 5). Contrasts of individual transgenic lines 308 and 310 vs. Col-0 are provided in Online Resource 16. In the −*ipd3* genotype of *Lotus *(*cyclops-4*), AMF exposure induced only 15 DEGs (Fig. 5A contrast A), while AMF treatment of +IPD3 Lotus induced 461 DEGs (Fig. 5A, contrast B). This is consistent with prior knowledge that AM symbiosis is strongly *IPD3-*dependent (Yano et al. 2008). In −*ipd3* Col-0 *Arabidopsis*, however, AMF *regulated* 497 DEGs (Fig. 5A contrast C), while AMF treatment of *+IPD3*^*Min*^* Arabidopsis regulated abundance of* only 3 transcripts (contrast D). In -AMF *Lotus* the presence of *IPD3* in *+IPD3 Gifu* produced 338 DEGs relative to −*ipd3 cyclops-4* (Fig. 5A contrast E), while the equivalent contrast in *Arabidopsis* produced 25 DEGs (contrast F). Contrast of the two genotypes under AMF treatment resulted in 38 DEGs for Lotus and 77 DEGs for Arabidopsis (Fig. 5A, contrasts G and H). This comparison showed that regulation of only 15 transcripts in Lotus were independent of IPD3 when Lotus roots were challenged with AMF in contrast to 497 genes that did not require IPD3^Min^ in Arabidopsis to be regulated in response to AMF inoculation. When we interpret these DEGs for the two Arabidopsis genotypes, it is important to keep in mind that the IPD3^Min^ protein is constitutively active and the transgene was controlled by the constitutive UBI10 promoter and therefore both highly expressed and active prior to the AMF-inoculation, unlike in *Lotus*. If IPD3-responsive genes were already regulated by constitutive IPD3^Min^ expression in *Arabidopsis*, further activation upon AMF exposure would not be expected.

**Fig. 6:**
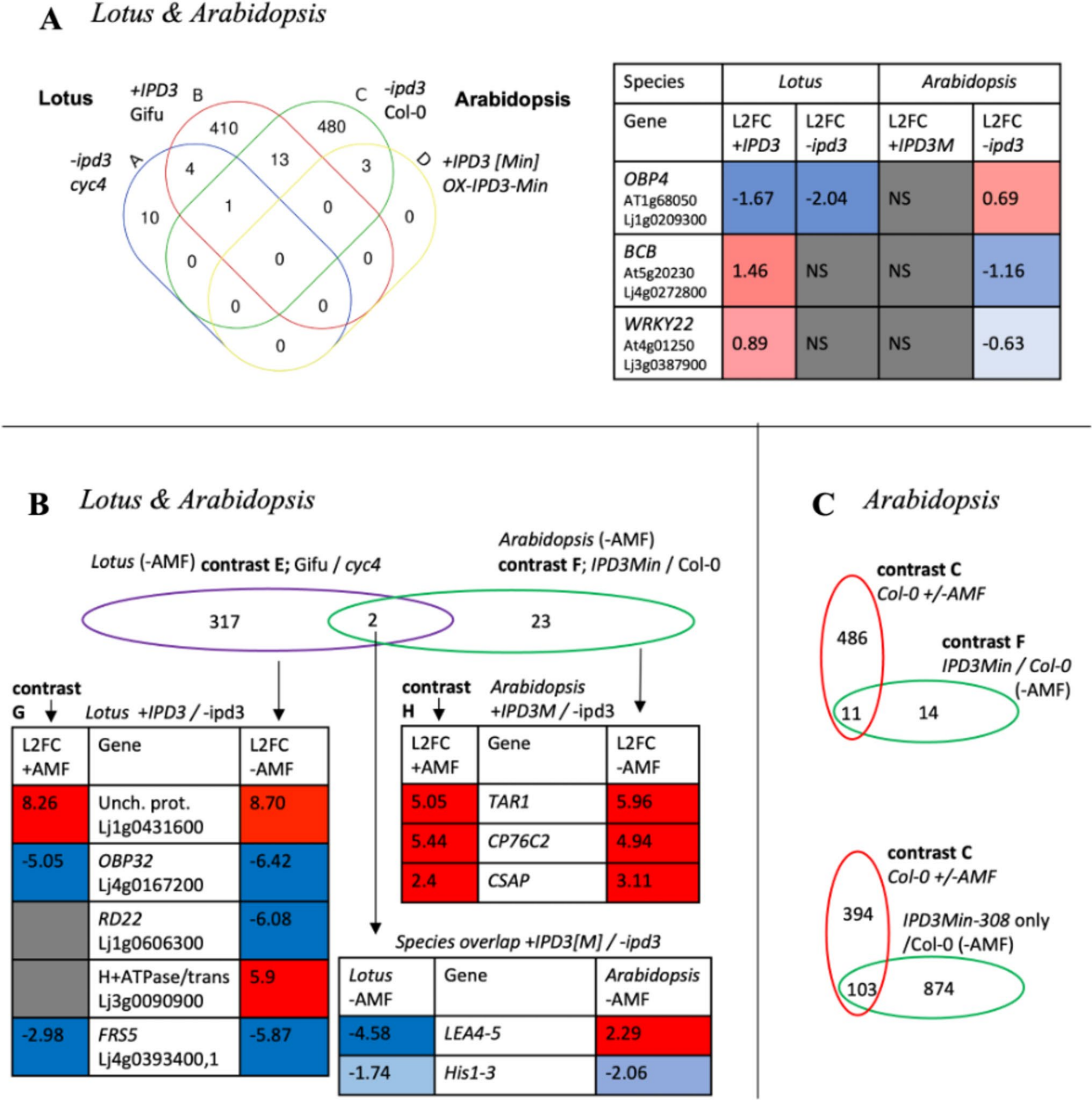
Cross-species comparison of AMF and *IPD3[Min]* responsive DEGs. **A** When the AMF-responsive DEGs within the respective +*IPD3* and −*ipd3* genotypes of *Lotus* and *Arabidopsis* are directly compared using annotated *Arabidopsis* homologs of *Lotus* genes, *OBP4* is shared by *−ipd3* Col-0 *Arabidopsis* with both *Lotus* genotypes, but oppositely regulated across species. Thirteen additional genes are shared by −*ipd3* Col-0 *Arabidopsis* and *+IPD3* Gifu *Lotus*, also with opposite regulation across species. **B** When the *IPD3-*responsive DEGs in non-AMF-treated *Lotus* and *Arabidopsis* are compared, *LEA4-5* and *His1-3* are shared across species, with *LEA4-5* being oppositely regulated. **C** When *IPD3-*responsive DEGs in non-AMF-treated *Arabidopsis* are compared to AMF-responsive DEGs in *−ipd3* Col-0 *Arabidopsis*, there is significant overlap. Eleven of 25 DEGs induced by *IPD3*^*Min*^ in both transgenic lines 308 and 310 are also induced by AMF in *−ipd3* Col-0. 103 out of 497 AMF-responsive DEGs (21%) in *−ipd3* Col-0 are also responsive to *IPD3*^*Min*^ in transgenic line 308, which has higher *IPD3*^*Min*^ expression and a larger overall DEG set. (See Online Resource 18)

Next, we asked if the genes that remained responsive to AMF in −*ipd3 Lotus* would be conserved in *Arabidopsis.* To detect candidate genes that are part of the *Lotus* AMF host response but independent of *IPD3*, we looked for overlap between AMF-responsive DEGs for both *Lotus* genotypes (Fig. 5B). Only 3 DEGs were shared: Lj1g0209300.1 (homolog of *OBF Binding Protein 4* (*LjOBP4*)); Lj2g0329400.1 (homolog of *Cinnamyl Alcohol Dehydrogenase 8* (*LjCAD8*)); and Lj5g0253700.1 (homolog of *Binding to TOMV RNA 1* (*LjBTR1*)). All of these genes were regulated in the same direction and at similar scale in both *Lotus* genotypes, suggesting they are elements of the native *Lotus* AMF response that are governed by an *IPD3-*independent mechanism. Despite *IPD3's* effect on the scale of AMF response in *Lotus, IPD3*^*Min*^ expression in *Arabidopsis* did not enable any new AMF-responsive DEGs, and the scale of response to AMF was much larger in the *−ipd3* Col-0 genotype than in *IPD3*^*Min*^ (Fig. 5C).

We then looked for overlaps in DEGs for AMF response between both genotypes of both species (Fig. 6). For direct cross-species comparisons *Lotus* genes were labeled with their closest *Arabidopsis* homologs as recorded in genome annotations; total DEG count for AMF response of Gifu was slightly reduced due to exclusion of 33 genes without an annotated *Arabidopsis homolog* (Online Resource 19)*.* One of the 3 *IPD3-*independent genes characterized in Fig. 5B as a member of the *IPD3*-independent *Lotus* AMF response, *OBP4* (*Lj1g0209300*; *At1g68050*), was also shared with the AMF response of Col-0 *Arabidopsis* (Fig. 6A). *OBP4* is a transcription factor that negatively regulates lateral root and root hair development in response to nitrate and abscisic acid (Ramirez‐Parra et al. 2017; Rymen et al. 2017; Xu and Cai 2019). Interestingly, *OBP4* was upregulated in the AMF response of *Col-0 Arabidopsis*, but downregulated in *Lotus*. *OBP4* was not differentially regulated in *IPD3*^*Min*^ plants.

In addition to *OBP4*, 13 genes were differentially regulated in response to AMF in Gifu *Lotus* and Col-0 *Arabidopsis*, but not *cyclops-4 Lotus* or *IPD3*^*Min*^* Arabidopsis* (Fig. 6A, Online Resource 19). In most of these genes, as for *OBP4*, the direction of regulation was reversed between species, including the next two most strongly regulated, multi-stress-responsive *Blue Copper Binding Protein* and pathogen response-related *WRKY22* (Fig. 6A). Consistent with defense effects of AMF observed in the network analysis, *WRKY22*, which was downregulated in *Arabidopsis*, is implicated in systemic acquired resistance and is characterized as a positive regulator of the SA pathway in SA-JA defense crosstalk (Kloth et al. 2016).

We also compared the effect of *IPD3* presence and absence across the two species in the absence of AMF treatment. Two genes differentially transcribed in +*IPD3 Lotus* relative to *−ipd3 Lotus* were also differentially transcribed in *+IPD3*^*Min*^* Arabidopsis* relative to *−ipd3* (Fig. 6B). Of these, osmotic stress responsive *Late Embryogenesis Abundant 4–5* was strongly downregulated by the presence of *IPD3* in *Lotus* but upregulated by the presence of *IPD3*^*Min*^ in *Arabidopsis*, while drought stress responsive *Histone H1-3* was downregulated in both species by the presence of respective *IPD3* versions.

Beyond specific genes, a shared and unexpected feature of the *+IPD3/−ipd3* contrast in both species was a pattern of many differentially expressed genes resulting from manipulation of IPD3 even in the absence of AMF (Figs. 5A, 6B). Many of the affected genes are related to biotic and abiotic stress. These included strong downregulation in *Lotus* of *Lj4g0393400.1* and *Lj1g0155200_LC.1,* both of which are homologs of *AtFRS5*, a gene involved in linking light perception to stress and pathogen defense via JA, and upregulation of *Lj4g0167200.1*, a homolog of the *AtJASSY* JA biosynthesis enzyme (Ma and Li 2018; Guan et al. 2019). The top most strongly regulated gene that was dependent on the presence of *IPD3* in *Lotus* was *Lj1g0431600.1*; its homolog *Lj1g0070100.1* was also upregulated, with an average L2FC of 7.14. These genes are homologous to an uncharacterized MYB/SANT transcription factor in *Arabidopsis*, *At2g24960.2*. In *Arabidopsis*, *IPD3*^*Min*^ expression also resulted in strong upregulation of genes related to JA pathogen response and auxin signaling including *Cytochrome P450 76C2* and *Tryptophan Aminotransferase Related 1* (Fig. 6B) (Lorenzo et al. 2003; Stepanova et al. 2008). Notably, both of these genes were included in module E of the network analysis, which had the strongest correlation to quantitative *IPD3*^*Min*^ expression (Online Resource 16).

While the differential response to AMF was nearly eliminated in *−ipd3 Lotus*, 337 Differentially Expressed Genes (DEGs) respond to *IPD3* in the contrast of *+IPD3* and *−ipd3 Lotus* without AMF inoculation (Fig. 5A). 135 of these DEGs resulting from knockout of *IPD3* in the absence of AMF treatment, were also found among DEGs resulting from AMF treatment of Gifu plants, accounting for 29% of the wildtype *Lotus* AMF response (Online Resource 19). Thus, although AMF-responsive upregulation of genes by IPD3 is known to be essential for normal AM function, its deletion also appears to partially replicate the AM response under the conditions of this experiment. This suggests that *IPD3* in *Lotus* may act as a context-dependent transcriptional repressor of a portion of its own targets.

A similar pattern is present in *Arabidopsis*, where 11 of 25 genes (44%) differentially expressed in response to *IPD3*^*Min*^ in the absence of AMF treatment were also induced by AMF treatment of Col-0 plants (Fig. 6C). These genes included, for example, the *FKF1* circadian clock gene identified in Fig. 5C as well as in the *IPD3-Min* responsive circadian clock module of the correlation network (Figs. 4, 5) (Online Resource 19). In transgenic line 308 where higher *IPD3*^*Min*^ expression corresponds to a larger absolute number of DEGs (Fig. 5A, Online Resource 9), this effect is much larger. The no-AMF contrast of *IPD3*^*Min*^*-308* vs Col-0 recapitulates differential regulation of 103 of the 497 (21%) AMF-responsive DEGs in Col-0 (Fig. 6C). These results confirm evidence in the correlation network that effects of *IPD3*^*Min*^ expression in *Arabidopsis* overlap partially with the AMF response*.* They indicate that while the nature of the response may differ*,* expression of constitutively activated *IPD3* in *Arabidopsis* can activate the transcriptional response to AMF even in the symbiont's absence, much as it does in AM host plants (Singh et al. 2014).

While the detailed comparisons of DEGs provide interesting insight into those genes and pathways that are apparently dependent on IPD3 activation in Lotus for a response to AMF challenge, the conservation of genes regulated by the constitutive expression of the DNA binding domain of IPD3 (IPD3^Min^) in Arabidopsis shows that there is still a set of genes regulated by this domain. While we used a conservative approach by only considering those DEGs that were regulated in both transgenic lines, the differential gene expression in the respective individual transgenic lines by IPD3^Min^ was much larger and can provide targets for further studies.

## Discussion

We successfully expressed *IPD3*^*Min*^, the DNA binding domain of the AM symbiosis-essential transcription factor IPD3, in the nonAM host plant *Arabidopsis* which lost both the AM trait and *IPD3* about 60–70 million years ago (Hohmann et al. 2015; Radhakrishnan et al. 2020). To understand the impact of *IPD3*^*Min*^ on remaining genetic pathways and biotic interactions, we compared the response of *Arabidopsis* genotypes to AMF inoculation, as well as the AM host plant *Lotus* and its *ipd3* mutant *cyclops-4*. Our results indicate that despite the long intervening period as a nonAM plant and the further loss of related genes, expressing *IPD3*^*Min*^ in *Arabidopsis* resulted in phenotypic and transcriptional effects. The preservation of molecular connections for *IPD3* raises the prospect of re-wiring nonAM plants to restore AM symbiosis, with applications in agriculture (French 2017; Lynch 2019). Although they are a phylogenetic minority, nonAM crops are responsible for almost half a billion metric tons of agricultural harvest every year (Hornstein 2022). With reported yield increases from mycorrhizae of 20–100% in various host crops, significant economic advantages could result if these benefits can be conferred on nonAM crops (Eo and Eom 2009; Pellegrino et al. 2015; Berruti et al. 2016)*.* In 2021, such an increase in US canola alone would have resulted in an additional 250,000–1.25 million metric tons of harvest worth 175–870 million dollars (USDA NASS 2022).

We also found evidence that *IPD3* may be subject to targeted regulation in *Arabidopsis*. Two full-length versions of *IPD3* transgenically expressed in *Arabidopsis* could not be detected at the protein level, while IPD3^Min^ was successfully expressed in roots and shoots (Figs. 2, S1). This suggests *IPD3* may be subject to silencing or degradation specific to the N-terminal portion excluded from IPD3^Min^. In prior work, we attempted to express *IPD3*^*Mt*^ and *IPD3*^*S50D*^ in the oilseed crop *Camelina sativa*, and observed low expression levels in multiple independent lines, consistent with evidence in the present study for silencing of these constructs in *Arabidopsis* (Hornstein 2022). We have also observed potentially deleterious effects for *IPD3*^*S50D*^ in *Camelina.* T1 transformants were repeatedly lost to severe fungal infections, and later generations produced unusual dwarfed growth phenotypes at nonmendelian ratios (Hornstein and Sederoff unpublished). It is possible that both the present study and past work in *Camelina* were biased by survivorship of T1 individuals which avoid a deleterious effect due to silencing.

Mechanisms for functional AM go beyond the CSP to essential plant functions including lipid biosynthesis, membrane and vesicle functions, nutrient transport, and defense (Wang et al. 2012; Behie and Bidochka 2014; Luginbuehl et al. 2017). Although symbiosis-specific genes that affect these functions in AM hosts have been lost in nonAM species, most belong to families with non-symbiosis-specific members that have similar molecular functions. In our experiment we observed *IPD3-*independent upregulation of lipid and 2-MAG biosynthesis genes in response to AMF in *Arabidopsis* (Figs. 3, 4)*.* Activation of 2-MAG biosynthesis via *RAM1* and *RAM2* is well-characterized as an essential feature of AM symbiosis that strictly depends on *IPD3* in host species (Wang et al. 2012; Gobbato et al. 2013; Pimprikar and Gutjahr 2018). In our results, 2-MAG-related transcription appeared to occur in response to AMF, but by action of related genes which are not symbiosis-specific (Yang et al. 2010, 2012b). Given that AMF was the only microbe tested, the biotic interaction terms and linkage to phosphate starvation as shown in the WGCNA and GOterm analysis may represent a generalized microbial response consistent with known effects in Arabidopsis (Finkel et al. 2019). In the context of engineering AM as an agriculturally useful trait, this raises the interesting possibility that non-symbiosis-specific genes retained in nonAM plants can be recruited to perform symbiotic functions (Hornstein and Sederoff 2023).

**Fig. 7:**
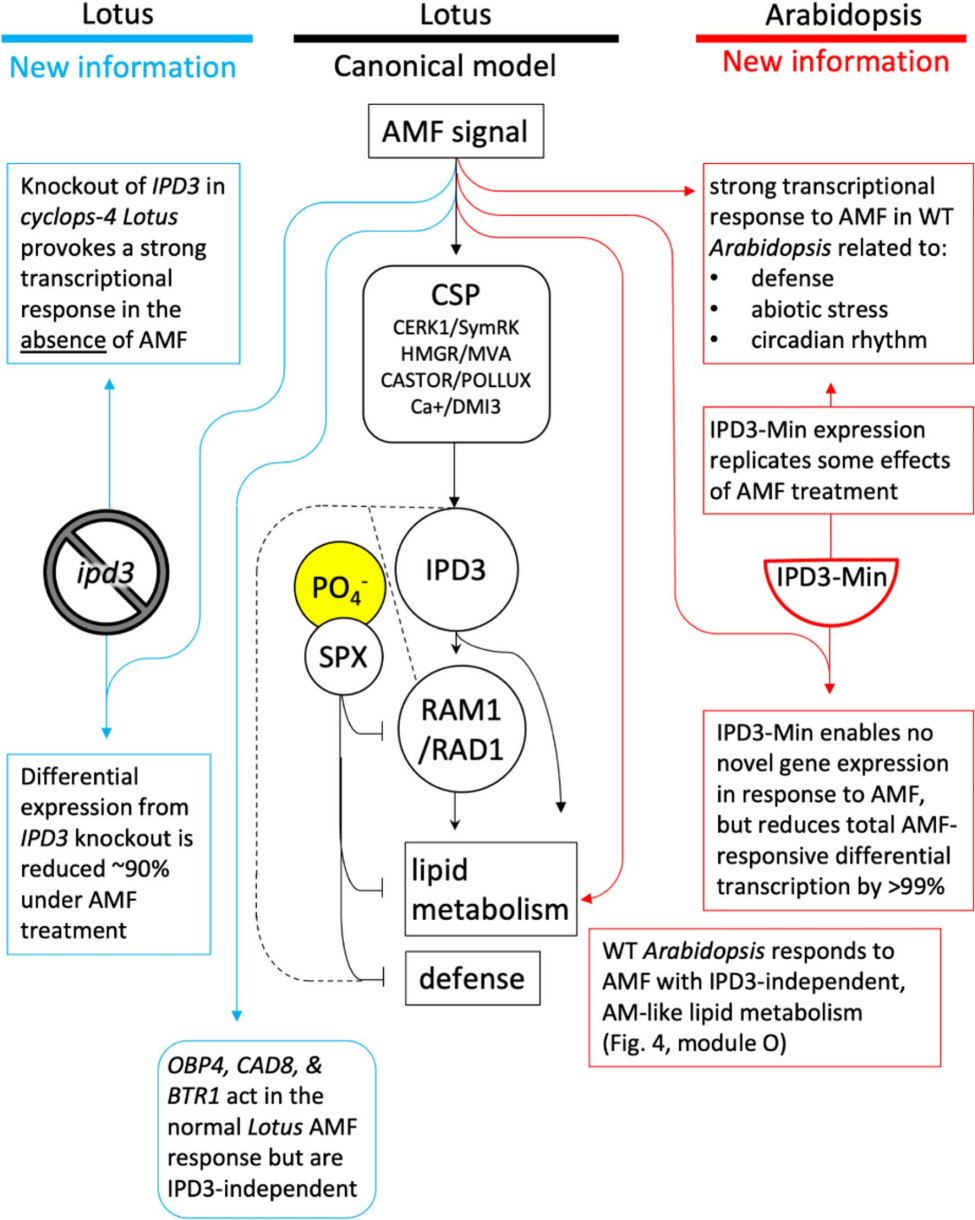
Novel roles for *IPD3* and responses to AMF in *Arabidopsis* and *Lotus*. In *Lotus*, we add to the current understanding of *IPD3* by showing that its knockout has a large effect even in the absence of AMF, and that the reduction in overall scale of AMF response in the *cyclops-4* genotype is due to a surprising partial replication of AMF-exposure-like transcription. In *Arabidopsis* we find evidence that AM-related lipid metabolism is altered by AMF independently of *IPD3* and despite the plant's nonAM status. Expression of *IPD3*^*Min*^ vastly reduced the amount of AMF-responsive differential expression in *Arabidopsis*, but not because *IPD3*^*Min*^ has little effect. *IPD3*^*Min*^ expression results in many differentially expressed genes in the absence of AMF, and the response to AMF in transgenic plants is small at least in part because many of the DEGs acting in the wild type *Arabidopsis* AMF response are similarly regulated in *IPD3*^*Min*^ transgenics even before AMF treatment is applied. Genes affected by *IPD3*^*Min*^ in *Arabidopsis* relate to functions including abiotic stress, pathogen defense, and circadian rhythm

Figure 7 lays out the main findings of this study in relation to the canonical knowledge of *IPD3*’s role in AM. In *Arabidopsis, IPD3*^*Min*^ plants showed a > 99% reduced transcriptional response to AMF compared to wild type (Fig. 5). This is in part because *IPD3*^*Min*^ expression places plants in an AMF-exposure-like transcriptional state prior to actual exposure to the fungus (Fig. 6C). This is reminiscent of results in symbiosis models where expression of phosphomimic *IPD3* leads to a constitutive symbiotic response in the absence of biotic stimulus (Singh et al. 2014; Gobbato 2015). Our results suggest that although the nature of the transcriptional response may differ, *IPD3*^*Min*^ acts analogously in *Arabidopsis* by activating the native AMF response even in the absence of the fungus. In *Lotus*, surprisingly, we found that −ipd3 knockout was associated with a similar priming effect. The AMF response in this *Lotus* genotype is ~ 90% reduced in our experiment, consistent with canonical understanding of *IPD3* (Yano et al. 2008; Pimprikar et al. 2016). However, in our experiment this was partly because a set of genes in uninoculated plants is regulated similarly upon *ipd3* knockout and AMF exposure (Fig. 5). This suggests IPD3 may have an unrecognized function as a direct or indirect repressor of some of its own targets.

The striking inverse effects in *Lotus* and *Arabidopsis* also highlight two limitations of our results for drawing comparisons between the two species and with prior work. First, the *IPD3* genotypes across the two species are not identical. The native *LjIPD3/CYCLOPS* gene in *Lotus* encodes a protein product that undergoes extensive protein-protein interactions and requires phosphorylation for activation, while the IPD3^Min^ protein we used in *Arabidopsis* is constitutively active (Yu et al. 2014; Singh et al. 2014; Pimprikar et al. 2016; Jin et al. 2016, 2018). Second, the effects we observed for AMF interaction may be specific to the early time point sampled in this study (48 h). We selected an early time point for the whole experiment because *Arabidopsis* cannot sustain AMF in monoxenic culture, but most studies allow interaction with AMF on the scale of weeks to establish mature colonization; earlier stages can involve rapidly shifting responses not seen later (Siciliano et al. 2007; Genre et al. 2008; Gutjahr et al. 2009; Handa et al. 2015; Nanjareddy et al. 2017; Prihatna et al. 2018). An idealized experiment might use *cyclops-4 Lotus* rescued by expression of *IPD3*^*Min*^, and a nurse-pot colonization system to support *Arabidopsis* AM colonization (Veiga et al. 2013; Fernández et al. 2019).

Prior studies in AM model plants at this early phase of colonization have found 20–650 DEGs in response to AMF treatment, germinated spore exudates, and various AM signal molecules at timepoints from 6 h to 48 days from contact (Czaja et al. 2012; Hohnjec et al. 2015; Giovannetti et al. 2015; Siciliano et al. 2007). Importantly, CSP gene knockout has previously been found to significantly alter the AMF (and nodulating rhizobacteria) response even at this early time point (Hohnjec et al. 2015; Høgslund et al. 2009). However, early-stage results in this experiment should also be viewed in recognition of the fact that late-stage colonization of AM host plants, as well as nurse-pot supported late-stage colonization of *Arabidopsis*, implicate much larger plant gene sets in the AM relationship (Fernández et al. 2019).

One area where *IPD3*^*Min*^ expression duplicates the effect of AMF treatment is downregulation of circadian clock genes that result in delayed flowering (Fig. 4) (Sawa et al. 2007; Para et al. 2007; Niinuma et al. 2008; Shim et al. 2017). This gene regulation may explain the delayed flowering phenotype in *IPD3*^*Min*^ transgenic *Arabidopsis* seen in our experiment if the root transcriptome effect extends to aboveground tissue (Figs. 1, S1). AM symbiosis affects flowering time in host plants, and the circadian clock is implicated in maintenance of the symbiosis itself as well as AM-mediated abiotic stress resistance (Hernandez and Allen 2013; Lee et al. 2019; Bennett and Meek 2020; Liu et al. 2022). In *Arabidopsis*, circadian rhythm genes are also known to act in microbiome construction, pathogen defense, development, and abiotic stress (Lee et al. 2005; Nakamichi et al. 2016; Newman et al. 2022; Xu et al. 2022; Singh 2022a, b). Remarkably, in mosses, the sole group of nonAM plants that retain CSP genes, *IPD3* specifically has been shown to mediate a stress-responsive reproductive transition (Kleist et al. 2022).

In uninoculated plants, two DEGs result from the respective *+IPD3/−ipd3* contrasts in both *Arabidopsis* and *Lotus*: *LEA4-5* and *HIS1-3* (Fig. 6B). Both of these genes are associated with response to abiotic stress including drought, heat, and cold (Dalal et al. 2009; Olvera-Carrillo et al. 2010; Rutowicz et al. 2015). *HIS1-3* is downregulated in the *+IPD3* genotype of both species, and *LEA4-5* is upregulated in *Arabidopsis* but downregulated in *Lotus* (Fig. 6).

A molecular function in stress response would subject *IPD3* to selection on factors unrelated to its role in AM symbiosis, and might help explain both the reasons for this gene’s loss in nonAM species, and its presence in charophyte ancestors of land plants long before the existence of AM symbiosis (Delaux et al. 2015; Delaux and Schornack 2021).

We also identified AMF and *IPD3-Min*-responsive downregulation of *Arabidopsis* genes involved in salicylic acid-mediated defense (Fig. 4C). Reduced transcription of *WRKY70*, *WRKY54*, and *PR5* by *IPD3*^*Min*^ in the absence of AMF suggests that plants expressing *IPD3*^*Min*^ have reduced baseline levels of SA-mediated defense to biotrophic pathogens (Glazebrook 2005; Blanco et al. 2005; Li et al. 2006; Yang et al. 2012a). Early AM colonization of host plants can involve a transient increased defense response followed by reduction, while *Arabidopsis* shows an early symbiosis-like response to AMF, followed by a strong defensive response under forced long-term interaction (Giovannetti et al. 2015) (Fernández et al. 2019; Cosme et al. 2021). While AM can confer resistance to pathogens and insects, genes acting in AM also enable infection by some pathogens (Wang et al. 2012; Siebers et al. 2016; Ried et al. 2019; Chen et al. 2021a; Dey and Ghosh 2022). The full mechanisms for such effects are not known, but comport with our evidence for perturbation of *Arabidopsis* defenses by a nominally AM-specific gene.

Recently, significant portions of the AMF genetic network were revealed to be under control of the phosphate-stress-responsive PHR-SPX system, which is conserved in *Arabidopsis* (Shi et al. 2021, 2022). In *Arabidopsis*, PHR also regulates the microbiome by reducing plant defenses when phosphate is low, resulting in recruitment of beneficial microbes (Finkel et al 2019; Martin-Rivilla et al. 2019; Castrillo et al 2017; Cho et al. 2013; Kotchoni and Gachomo 2006; Dangl 1998). Given knowledge that both AM and non-AM microbial relations are part of the PHR-regulated phosphate response network, it is possible that the *IPD3*^*Min*^ effect in our experiment relates to conserved, non-AM-specific points of crosstalk with defense and symbiosis (Fig. 7).

Regardless of whether plants are formally labeled non-hosts in isolation, in natural settings they continue to interact with ubiquitous AM fungi and can display partial and transitional phenotypes (Ma et al. 2018; Cosme et al. 2018). It is not surprising that an ancient, conserved trait is interconnected to other aspects of plant life. Our results show that despite the loss of genes essential for AM symbiosis, connections to this trait remain in place in *Arabidopsis*, and can be highlighted, even re-activated by expression of *IPD3*^*Min*^. The transcriptional effects we identified suggest specific targets for follow-up studies to directly assess pleiotropic effects, including pathogen sensitivity and abiotic stress resilience.

### Supplementary Information

Below is the link to the electronic supplementary material.

Supplementary file1 (DOCX 33 KB)
Online Resource 1 Supplemental experimental procedures providing further protocol detail for bioinformatic and laboratory analysis

Supplementary file2 (PNG 3327 KB)
Online Resource 2 Comparison of IPD3 protein versions used in this study and validation of RNA and protein expression in transgenic plants. The +C control sample is a synthetic peptide of the 349 C-terminal residues of MtIPD3 with expected size 39.98 kDa; expected size of IPD3-Min is 29.19 kDa and expected size of full-length IPD3 versions is 57.98 kDa. (A) Protein sequence alignment of Medicago truncatula IPD3, S50D phosphomimic IPD3, constitutively active DNA binding domain IPD3Min, Lotus japonicus IPD3/CYCLOPS, and the truncated C-terminal fragment resulting from the cyclops-4 mutation in Lotus. (B) Schematic of protein domains as affected by variants in this study. (C) Western blot of 3 IPD3 versions from transgenic Arabidopsis roots and shoots. This blot was visualized with fluorescent secondary antibody under 488/530 nm excitation/emission conditions (see Methods and Online Resource 1), causing the target protein bands to appear as light areas of high exposure while protein size ladder bands are visualized by dye and appear dark.(D) rt-PCR to confirm expression of IPD3 versions as RNA in leaves of T3 individuals.

Supplementary file3 (XLSX 1185 KB)
Online Resource 3 Shotgun proteomic quantification data of IPD3 and native proteins in Arabidopsis. Protein abundance is quantified as Normalized Spectral Abundance Factor (NSAF) according to Zybailov et al (2006), which is the number of unique protein fragments mapped to a specific reference sequence (spectral counts, SpC) divided by the total length of that protein (L), divided by the sum of the latter across all proteins present in the dataset (e.g. for protein n, [(SpCn)/(Ln)]/Σ[(SpC)/(L)]. NSAF is therefore a positive metric between 0 and 1, and the sum of NSAF for all proteins in a sample always adds up to 1, with the protein with highest NSAF having the highest estimated abundance. Note that matches of IPD3Mt and IPD3S50D are detected in the IPD3Min transgenic line due to the fact that IPD3Min is identical to the C-terminal formation of those reference sequences. No protein sequence originating from the N-terminal portion of IPD3 was detected in IPD3Min plants, and no IPD3 protein sequence of any kind was detected in plants transformed with IPD3Mt or IPD3S50D.

Supplementary file4 (PNG 3488 KB)
Online Resource 4 Mature phenotypes of IPD3-Min transgenic and control Arabidopsis lines in T3. (A-E) Transgenic lines 303, 308, 310, 312, 357, respectively; (F) wild type Col-0; (G) null segregants of transgenic line 308. Red line in all pictures shows 30 cm from surface

Supplementary file5 (XLSX 32 KB)
Online Resource 5 Raw phenotypic data and statistical output for Arabidopsis phenotyping experiments

Supplementary file6 (PNG 5009 KB)
Online Resource 6 Representative root images of transgenic and control lines. In an original set of experiments, the roots of soil-grown IPD3Min transgenic plants were bright pink while wild type and null segregant controls were white, and IPD3Mt and IPD3S50D were faint pink to white. In a followup experiment, multiple independent empty vector controls containing the mCherry marker also ranged from pink to white. Red stars mark those root images visually assessed to show any degree of pink coloration.

Supplementary file7 (PNG 1262 KB)
Online Resource 7 Images of 5-week-old Lotus japonicus seedlings of Gifu wildtype and cyclops-4 knockout mutant of ipd3 as grown on sterile petri dishes for the transcriptome experiment. Scale bar = 2 cm (scale tiles within image are denoted in cm)

Supplementary file8 (PNG 455 KB)
Online Resource 8 Principal component analysis shows clustering of transcriptomes by IPD3 genotype and AMF treatment in two species. (A) Lotus Gifu samples cluster according to AMF treatment along PC1. Gifu and cyclops-4 samples cluster by genotype along PC2. In contrast to Gifu, cyclops-4 plants do not separate along PC1 according to AMF treatment; both treatment groups instead cluster near AMF-treated Gifu plants along this axis. (B) Arabidopsis samples cluster by genotype along PC1 (IPD3M = IPD3Min transgenic). While wild type Col-0 plants cluster by AMF treatment along PC2, there is no equivalent separation by AMF treatment within either IPD3Min line (308; 310)

Supplementary file9 (PNG 172 KB)
Online Resource 9 IPD3-Min and mCherry transcript level in Arabidopsis transcriptome samples. Average IPD3-Min transcript count in line 308 is 36,591, ~4X higher than line 310 with average IPD3-Min count of 9,250

Supplementary file10 (PNG 181 KB)
Online Resource 10 Principal Component Analysis of Arabidopsis transcriptomes under low-nutrient conditions with AMF treatment. Only IPD3Min transgenic line 308 was used for the low-nutrient experiment. Transcriptomes cluster by IPD3 genotype along PC1, which accounts for 62% of variation

Supplementary file11 (PNG 672 KB)
Online Resource 11 Heatmap of complete gene expression correlation network (WGCNA) with module-trait correlations. Cells included in figure 3 of the main text are highlighted in yellow. The top number in each cell is the signed eigengene correlation of that module to value of trait shown on the X axis. A value of 1 indicates perfect positive correlation, -1 would indicate perfect inverse correlation. Bottom value shown in parentheses is the P-value of that module-trait correlation. Trait/treatment labels are as follows: Nutrient Level: determined by medium composition as described in Methods, positive correlation of a module to Nutrient Level indicates positive correlation of gene expression for module members with combined macronutrient concentration; +/-AMF: determined by presence or absence of germinated AM fungus spore treatment, positive correlation of a module to +/-AMF indicates positive correlation of gene expression for module members with presence of AMF; Transgenic Line: determined by transgenic genotype, discriminating between independent IPD3Min lines 308 and 310. Positive or negative correlation to Transgenic Line indicates that gene expression for that module is affected by transgenic genotype but is non-quantitative (see following); IPD3-Min Expression: determined by quantitative transcript level of IPD3Min, agnostic of line identity. Positive correlation of a module with IPD3-Min Expression indicates positive correlation of gene expression for module members with level of IPD3Min expression across all samples. Correlation of a module to Transgenic Line but not IPD3-Min Expression (i.e. Modules B, I, P), or correlation in opposite directions (not observed), would suggest that effects in that module could be due to insertion site effects rather than IPD3Min expression itself; mCherry Expression: determined by quantitative transcript level of mCherry marker gene across all samples.

Supplementary file12 (XLSX 15519 KB)
Online Resource 12 WGCNA gene module membership and trait correlations in Arabidopsis

Supplementary file13 (PDF 156 KB)
Online Resource 13 Semantic similarity clustering of gene ontology (GO) terms of all modules shown in figure 3 of the main text (including all Arabidopsis modules significantly correlated to IPD3Min expression). Size of the point on the plot for each GO term corresponds to its fold enrichment in that module, and intensity of color corresponds to FDR-corrected p-value of the enrichment. Proximity of the points represents their semantic similarity as determined by ReviGO (Supek et al 2011) from relatedness and frequency within the Arabidopsis gene ontology reference

Supplementary file14 (XLSX 65 KB)
Online Resource 14 Gene Ontology enrichment of Arabidopsis network modules

Supplementary file15 (XLSX 7511 KB)
Online Resource 15 Transcript count data of Arabidopsis

Supplementary file16 (XLSX 463 KB)
Online Resource 16 Gene sets resulting from differential expression contrasts summarized in figure 5

Supplementary file17 (PNG 404 KB)
Online Resource 17 Gene co-regulatory modules resulting from WGCNA analysis of Lotus transcriptomes. AMF corresponds to AM fungus treatment. Genotype reflects the presence of functional IPD3 in the wild type with a value of +1 and its absence in the cyclops-4 mutant with a value of 0

Supplementary file18 (PNG 392 KB)

Supplementary file19 (XLSX 27421 KB)
Online Resource 18 WGCNA gene module membership and trait correlations in Lotus.

Supplementary file20 (XLSX 396 KB)
Online Resource 19 Cross-species comparisons of DEGs summarized in figure 6

## Data Availability

Transcriptome data is publicly available via GEO accession number GSE225213, https://www.ncbi.nlm.nih.gov/geo/query/acc.cgi?acc=GSE225213. Proteomics data have been deposited to the ProteomeXchange Consortium via PRIDE identifier PXD040665 (Reviewer access at https://www.ebi.ac.uk/pride/login with Username: reviewer_pxd040665@ebi.ac.uk and Password: CVDhdrfh).
