# Supplementary material for: IPD3, a master regulator of arbuscular mycorrhizal symbiosis, affects genes for immunity and metabolism of non-host Arabidopsis when restored long after its evolutionary loss
Source: Plant Mol Biol. 2024 Feb 18;114(2):21. doi: 10.1007/s11103-024-01422-3 (PMC10874911; doi:10.1007/s11103-024-01422-3)
Supplement: Supplementary file 1 — Supplementary file1 (DOCX 33 KB) Online Resource 1 Supplemental experimental procedures providing further protocol detail for bioinformatic and laboratory analysis [file 11103_2024_1422_MOESM1_ESM.docx]

Supplemental experimental procedures

Assembly of transgenic constructs

Primer pairs for cloning and screening are shown in the table contained within this section. The coding sequence of *Medicago truncatula IPD3* (Genbank accession EF569224.1)^1^ was synthesized including restriction sites for *BamHI* (5') and *SphI* (3') (Integrated DNA Technologies, Research Triangle Park, NC) and assembled into pUC19 (Addgene plasmid #50005) using respective enzymes and T4 ligase (NEB, Ipswich, MA), followed by transformation and selection with carbenicillin. The *S50D-IPD3* coding sequence was constructed by restriction-ligation of pUC19-*MtIPD3* with a synthesized fragment containing the first 781 bases of *IPD3* including the S50D mutation (IDT, Research Triangle Park, NC) via *Pst1* and *BamHI* (NEB, Ipswich, MA).

Plant expression constructs were prepared in the pCAMBIA0380 (Genbank AF234290.1) backbone by seamless assembly with the NEB Hi-Fi cloning kit (NEB, Ipswich, MA). Amplifications for Gibson assembly used Superfi Platinum II polymerase (Invitrogen, Waltham, MA). Assembly fragments were digested with *DpnI* and gel-purified with the NEB Monarch gel extraction kit (NEB, Ipswich, MA). Plasmids were transformed into *E. coli*, recovered and plated onto LB with appropriate antibiotics. Colonies screened by direct PCR were used to inoculate liquid culture and plasmid was purified from 3-100 mL of saturated culture using Qiaprep Spin Miniprep or Zymo Midiprep kits and Sanger-sequenced for confirmation, including plasmids produced as intermediate cloning steps (Qiagen, Germany; Zymo, Irvine, CA).

First, the 2X35S:mCherry marker sequence amplified from pC-GW-mCherry (Genbank KP826771.1) ^2^ was assembled into the empty marker site of pCAMBIA0380 to produce pC0380-MC using primer pairs 1 and 2. Next, a synthetic DNA fragment containing the 35S promoter, *Nopaline Synthase* terminator (*tNOS*), and *Arabidopsis* *Heat Shock Protein* terminator (*tAtHSP*), was then assembled into the multiple cloning site (MCS) of pC0380-mCherry linearized with primer pair 3 to produce pC0380-MC-35S (Nagaya et al. 2010). The 2 kb *Arabidopis Ubiquitin 10* promoter (*pAtUBQ10*) was amplified from Col-0 *Arabidopsis* genomic DNA with primer pair 4 and assembled in pC0380-MC-35S linearized with primer pair 5 to produce pC0380-MC-UBQ, in which *pAtUBQ10* replaces *p35S* for the gene of interest^3^. The purpose of the *tNOS:AtHSP* double terminator was to increase expression and the purpose of replacing *p35S* with *pATUBQ10* was to provide a promoter with expression document in multiple root tissues as described by Nagaya et al and Ivanov and Harrison, respectively^3,4^.

*MtIPD3* or *S50D-IPD3* coding sequences were amplified from respective pUC19 cloning plasmids with primer pair 6 and assembled in position following *pAtUBQ10* in pC0380-MC-UBQ linearized with primer pair 7. *IPD3-Min* was cloned by amplifying the N-terminal DNA-binding domain (aa 254-513) as described in Singh et al^5^ from *pUC19-MtIPD3* with addition of a 5' start codon using primer pair 8, and assembled in pC0380-MC-UBQ.

Plant transformation

Col-0 *Arabidopsis* were transformed by the direct-dip protocol as described by Davis et al. ^6^. Briefly, *Agrobacterium tumefaciens* GV3101 transformed with expression constructs was grown to saturation in yeast extract-beef medium with addition of 2.5% sucrose and appropriate antibiotics and screened by colony PCR with primer pair 9 or 10 to confirm presence of the trangene. An additional 2.5% sucrose and 300 uL/L Silwet L-277 (Phytotech Labs, Lenexa, KS) were added to bacterial cultures and flowering *Arabidopsis* were dipped directly into this mixture; bacteria were not resuspended in transformation buffer and vacuum was not used. Plants were covered in the dark for 24 hours then returned to growing conditions; transformation was repeated 3 times, 4-6 days apart.

T1 *Arabidopsis* seed were screened by mCherry fluorescence, and sequence of the transgene was confirmed by CTAB DNA extraction, PCR with primer pairs 9 and/or 10, and Sanger sequencing^7^. RNA was extracted from leaves using the Invitrogen Purelink RNA Mini kit (Invitrogen, Waltham, MA), treated with the Turbo DNA-free DNAse kit (Invitrogen, Waltham, MA) and screened for expression of the transgene by PCR of cDNA generated with the Takara EcoDry cDNA kit (Takara Bio, Japan). Lines were brought to homozygosity over 3 generations, with PCR and Sanger sequence confirmation of the transgene in each generation.

| PCR primers used in screening and assembly | | | |
| --- | --- | --- | --- |
| Pair /set | Primer | Sequence 5’🡪3’ | Target/purpose |
| 1 | EL309 | GCTCTAGCCAATAGAATATAAATTGGGAGCT-GAATGCCACCGTC | Linearization of pCAMBIA 0380 with removal of marker And overlap sequences for product of pair 2 |
|  | EL310 | AAAATCCAGATCCCTAGATCGGCGCGCCGGG |  |
| 2 | EL311 | GCGCCGATCTAG-GGATCTGGATTTTAGTACTGG | mCherry marker with overlap sequences for product of pair 1 |
|  | EL312 | CAATTTATATTC-TATTGGCTAGAGCAGCTTG |  |
| 3 | EL293 | GGAATTAAACTATCAGTGTTTGACAG | linearization of pCAMBIA 0380 at MCS |
|  | EL294 | CGATCAATCACCGCGTCAATAAG |  |
| 4 | EL385 | ATTGACGCGGTGATTGATCGAGTCTAGC-TCAACAGAGCTTTT | Amplification of pATUBQ10 with overlaps for pair 5 product |
|  | EL386 | TTCCTTATATAGAGGAAGGGGACAAAT-TCGATCGCACAAACTAG |  |
| 5 | EL387 | TCTAGTTTGTGCGATCGAATCCCTTCCT-CTATATAAGGAA | pC0380-MC-*p35S::tNOS:tAtHSP* linearization with removal of p35S and overlaps for pair 4 product |
|  | EL388 | AAGCTCTGTTGAGCTAGACTCGATCAAT-CACCGCGTCAATAAG |  |
| 6 | EL357 | CCCTTCCTCTATATAAGGAAATGGA-AGGGAGAGGATTTTC | MTIPD3 or S50D-IPD3 with overlaps for product of pair 7 |
|  | EL358 | GCCAAATGTTTGAACGATCG-TCAAATCTTTCCAGTTTCTG |  |
| 7 | EL359 | CAGAAACTGGAAAGATTTGA-CGATCGTTCAAACATTTGGC | pC0380-MC-*pUBQ::tNOS:tAtHSP* linearized at GOI site with overlaps for product of pair 6 or 8 |
|  | EL360 | GAAAATCCTCTCCCTTCCATTTCCTT-ATATAGAGGAAGGG |  |
| 8 | EL361 | CCCTTCCTCTATATAAGGAAATGGA-GAAAGAAGCTGCAGAAGA | IPD3-min with addition of start codon and overlap for product of pair 7 |
|  | EL360 | GAAAATCCTCTCCCTTCCATTTCCTT-ATATAGAGGAAGGG |  |
| 9 | EL391 | CTATAAAACAATACCCAAAGAGCTC | Amplifies full length transgene with partial flanking sequences of *pUBQ10* and *tHSP* |
|  | EL482 | GTTATATGCTGCAGAAGAGATCC |  |
| 10 | EL369 | ATGGAGAAAGAAGCTGCAGAAGA | screening fragment for All *IPD3* versions |
|  | EL370 | TCAAATCTTTCCAGTTTCTG |  |

Protein analysis

For Western blotting, tissue was frozen in liquid nitrogen and ground in a mortar and pestle. Extraction buffer consisting of 50 mM Tris-HCL pH 8, 150 mM NaCl, .1 mM Brij-35, 1 mM EDTA, 2.5 mM DTT, 2% SDS w/v, 10% glycerol v/v, and freshly added 2% 2-mercaptoethanol v/v and 1.5% Sigma plant protease inhibitor cocktail (Sigma-Aldrich, Burlington, MA) was added to the frozen tissue and ground until thawed. Samples were centrifuged at 4C for 10 minutes, and the supernatant was diluted 1:1 in NuPage LDS sample buffer (Invitrogen, Waltham, MA) with an additional 2% v/v 2-mercaptoethanol and heated at 95C for 10 minutes. 45 uL of sample was run on a Novex Wedgewell 12% Tris-Glycine (Invitrogen, Waltham, MA) acrylamide gel in SDS running buffer for approximately 25 minutes at 225 mV. Protein was transferred to PVDF membranes using an iBlot (Thermo Fisher, Waltham, MA). Membranes were blocked in Tris-buffered saline plus 2% BSA and 4uL/mL Tween-20 for 1 hour, then incubated for at least 16 hours at 4C with 1:1000 dilution of custom rabbit anti-IPD3 peptide primary polyclonal antibody (Genscript, China) in blocking buffer. The antibody was generated to a synthetic peptide comprised of the 349 C-terminal peptides of IPD3, excluding the N-terminal portion of IPD3 up to position 50 in order to avoid potential selectivity between the IPD3^Mt^ and IPD3^S50D^ sequence variants present in different transgenic lines in this study. When later used as a positive control for Western blots, the expected size of the positive control (39.98 kDa) therefore varied from the expected size of both IPD3^Mt^/IPD3^S50D^ ( 57.98 kDa) and IPD3^Min^ (29.19 kDa). Membranes were washed 3 times in blocking buffer, then incubated at RT in TBS-T with 1:2,500 dilution of donkey anti-rabbit Alexa Fluor 488-conjugated fluorescent secondary antibodies (Thermo Fisher, Waltham, MA) for 2-4 hours. Blots were imaged on a GelDoc SR (Bio-Rad, Hercules, CA).

For shotgun proteomics, ground leaf and root tissue was used for protein extraction. Five biological replicates were included for each line. We used 200-300 mg of hand-ground tissue from each sample for protein extraction in 1 ml of SDT lysis buffer [4% (w/v) SDS, 100 mM Tris-HCl pH 7.6, 0.1 M DTT]. We lysed the ground tissue by bead-beating in lysing matrix E tubes (MP Biomedicals) with a Bead Ruptor Elite (Omni International) for 5 cycles of 45 sec at 6.45 m/s with 1 min dwell time between cycles; followed by heating to 95°C for 10 min. The lysates were centrifuged for 5 min at 21,000 x g to remove cell debris. Supernatant was used for purification and digestion using the filter-aided sample preparation (FASP) protocol described by Wisniewski et al.^8^. All centrifugations mentioned below were performed at 14,000 x g. Samples were loaded onto 10 kDa MWCO 500 μl centrifugal filters (VWR International) by combining 60 μl of lysate with 400 μl of Urea solution (8 M urea in 0.1 M Tris/HCl pH 8.5) and centrifuging for 20 min. This step was repeated once to load a total of 120 ul of lysate. Filters were washed once by applying 200 μl of urea solution followed by 20 min of centrifugation to remove any remaining SDS. 100 μl IAA solution (0.05 M iodoacetamide in Urea solution) was added to filters for a 20 min incubation at room temperature followed by centrifugation for 20 min. The filters were washed three times with 100 uL of urea solution and 20 min centrifugations, followed by a buffer exchange to ABC (50 mM Ammonium Bicarbonate). Buffer exchange was accomplished by three cycles of adding 100 μl of ABC and centrifuging for 20 min. Tryptic digestion was performed by adding 1 μg of MS grade trypsin (Thermo Scientific Pierce, Rockford, IL, USA) in 40 μl of ABC to each filter and incubating for 16 hours in a wet chamber at 37°C. Tryptic peptides were eluted by adding 50 μl of 0.5 M NaCl and centrifuging for 20 min. Peptide concentrations were determined with the Pierce Micro BCA assay (Thermo Scientific) following the manufacturer’s instructions.

For analysis of specific protein size ranges we used SDS-PAGE followed by in-gel digestion of specific bands. We used the proteomics lysate in SDT buffer generated for the shotgun proteomic approach also for SDS-PAGE. Only one replicate of each transgenic line was used for SDS-PAGE. We mixed lysate containing 30-40 µg of protein with Laemmli buffer, and heated to 95°C for 5 min prior to loading. SDS-PAGE was done using a 12% separating gel with a 5% stacking gel that was run at 80 V for 30 min and then 120V for 1 hour and 15 min. The gel was fixed with 40% EtOH and 10% acetic acid for 30 min followed by staining with QC Colloidal Coomassie stain (Bio-Rad) overnight. Gel pieces corresponding to target protein sizes were excised and in-gel digestion was performed according to Shevchenko et al.^9^. Gel pieces were destained in 40 mM ammonium bicarbonate buffer with 50% acetonitrile. Reduction was performed using 20 mM DTT for 30 min at 56°C and alkylation was performed using 55 mM iodoacetamide for 20 min at room temperature. In-gel digestion was performed overnight using a 0.02 µg/µl trypsin solution. Elution of peptides was done by addition of 100% acetonitrile followed by a second elution using 50% acetonitrile and 5% formic acid. The peptide mixture was dried in a speedvac and rehydrated using 10 µl 2% formic acid.

Shotgun proteomics samples were analyzed by 1D-LC-MS/MS as described in Mordant and Kleiner ^10^. The samples were blocked by treatment. Nontransgenic control samples were run first to avoid any false detections due to minimal amounts of carryover of peptides from the transgenic plant samples to control samples. We loaded of 1.4 μg peptide of each sample with an UltiMate^TM^ 3000 RSLCnano Liquid Chromatograph (Thermo Fisher Scientific) in loading solvent A (2% acetonitrile, 0.05% trifluoroacetic acid) onto a 5 mm, 300 µm ID C18 Acclaim® PepMap100 pre-column (Thermo Fisher Scientific). Elution pre-column and separation of peptides on the analytical column (75 cm x 75 µm analytical EASY-Spray column packed with PepMap RSLC C18, 2 µm material, Thermo Fisher Scientific; heated to 60 °C) was achieved using a 140 min gradient going from 95 % buffer A (0.1 % formic acid) to 31 % buffer B (0.1 % formic acid, 80 % acetonitrile) in 102 min, then to 50 % B in 18 min, to 99 % B in 1 min and ending with 99 % B. The analytical column was connected to an Orbitrap Eclipse Tribrid mass spectrometer (Thermo Fisher Scientific) via an Easy-Spray source. Eluting peptides were ionized via electrospray ionization (ESI). Carryover was reduced by two wash runs (injection of 20 µl acetonitrile, 99 % eluent B) between sample blocks. MS1 spectra were acquired in the Orbitrap by performing a full MS scan at a resolution of 60,000 on a 380 to 1600 m/z window. MS2 spectra were acquired using a data dependent approach by selecting for fragmentation the 15 most abundant ions from the precursor MS1 spectra. We used a normalized collision energy of 27 for HCD in the ion-routing multipole to generate the peptide fragments for MS2 spectra. Other settings of the data-dependent acquisition included: a maximum injection time of 50 ms, a dynamic exclusion of 25 sec and exclusion of ions of +1 charge state from fragmentation. About 50,000 - 60,000 MS/MS spectra were acquired per sample.

For measurement of gel bands after in-gel digest, 10 µl of peptide mixture was injected using the same Liquid Chromatograph and protocol as above. The analytical column was connected to an Orbitrap Exploris 480 mass spectrometer (Thermo-Fisher Scientific) with the same MS1 and MS2 parameters used on the Eclipse. About 95,000 MS/MS spectra were acquired per sample.

Protein identification and quantification

A database containing all protein sequences from *A. thaliana* cv. Col-0 (Uniprot:UP000006548), as well as the IPD3 vector sequences was used. Sequences of common laboratory contaminants were included by appending the cRAP protein sequence database (<http://www.thegpm.org/crap/>). The final database contained 39,446 protein sequences and is included in the PRIDE submission (see data access statement) in fasta format. Searches of the MS/MS spectra against this database were performed with the Sequest HT node in Proteome Discoverer version 2.3.0.523 (Thermo Fisher Scientific) as described in Mordant and Kleiner ^10^. Peptide false discovery rate (FDR) was calculated using the Percolator node in Proteome Discoverer and only peptides identified at a 5% FDR were retained for protein identification. Proteins were inferred from peptide identifications using the Protein-FDR Validator node in Proteome Discoverer with a target FDR of 5%. The data were normalized by calculating normalized spectral abundance factors (NSAFs) according to Zybailov et al.^11^ and multiplied by 100 to represent the relative protein abundance as a percentage.

Growth phenotyping

Plants were grown in the NCSU phytotron under long day conditions in 8 oz pots filled with SunGro propagation mix (Sungro, Agawam, MA). Pots were hand-watered daily with deionized water and were not fertilized. Plants were censused daily for onset of bolting, onset of flowering, and duration of seeding. Mature plants were dried for 1 week, then seeds were manually harvested and hand-cleaned to remove chaff before weighing.

Anthocyanin extraction

To check for anthocyanins and carotenoids in visibly red roots of transgenic plants, roots of mature soil-grown plants were briefly washed, then frozen with liquid nitrogen and hand-ground. 600 uL of pure methanol, 60% methanol/water v/v + 1% HCl, or 80% acetone was added to ~100 mg of ground tissue and further ground with a plastic pestle in a 1.7 mL microtube, then centrifuged for 10 minutes at 16,000 x g, and supernatant harvested. Absorbance was measured at 10 nm intervals using a Synergy HT plate reader (Biotek, Winooski, VT).

Transcriptome experiment preparation

Plants were grown on sterile petri dishes containing 50 mL of either 1/2MS or low-nutrient MS (LN) media. LN media was prepared from 20X macronutrient solution and MS micronutrient solution (Phytotech Labs, Lenexa, KS) as described in the table below. Plants were separated from growing medium by a 30 uM nylon mesh (Genesee, RTP NC) to prevent roots from growing into the media. Plates were covered with black cardboard sleeves to prevent light exposure of roots, and held at a 60 degree angle during growth in a growth chamber under long-day conditions (16 hours light/8 hours dark). *Lotus* seeds were treated identically to *Arabidopsis* with the exception of being manually scarified on 300-grit sandpaper prior to sterilization.

| Ingredients and macronutrient concentrations of nutrient media used in this study along with preprepared micronutrients at standard ½ MS concentration. | | | | |
| --- | --- | --- | --- | --- |
| Reagent | Macronutrient | mM in ½ MS | mM in LNMS | LN as % of 1/2MS |
| KH2PO4 | -- | 0.625 | 0 | -- |
| K2HPO4 | -- | 0 | 0.0625 | -- |
| NH4NO3 | -- | 10.3 | 2 | -- |
| KNO3 | -- | 9 | 2 | -- |
| MgSO4*7H2O | -- | 0.75 | 0.75 | -- |
| CaCl2 | -- | 1.5 | 1.5 | -- |
| -- | NH4 | 10.3 | 0.2 | 0.97% |
| -- | NO3 | 19.3 | 0.1 | 1.04% |
| -- | total N | 29.6 | 0.3 | 1.01% |
| -- | PO4 | 0.625 | 0.003 | 0.50% |
| -- | K | 9.625 | 0.11 | 1.10% |

500 uL of AMF inoculum containing ~200 aseptic *R. irregularis* spores (Premier Tech, Canada) germinated at 26C for 1 week prior to use was pipetted onto the roots of each plant. Control plants were mock-inoculated with sterile water. Plants were allowed to sit horizontally for 2 hours after inoculation, then returned to the growth chamber for the remainder of the 48 hours prior to collection. All treatments and tissue collection were completed between 4 and 6 hours from the start of the light period, and roots were immediately frozen in liquid nitrogen. Each replicate consisted of the pooled roots of 5 seedlings from the same plate.

RNA was extracted and DNAse treated as described previously, then sequenced by BGI Group (China). RNA libraries were prepared at BGI using a strand-specific, poly-A enrichment method and then sequenced on the DNBSEQ platform to obtain 100 bp paired-end reads. Raw reads were filtered for adaptor contamination and low-quality sequences using SOAPnuke^12^ and remaining sequences were evaluated using FASTQC^13^ to ensure only high-quality read pairs (Q>30) were used for downstream analysis.

Read alignment was performed using BBSplit, an aligner designed for metagenomics within the BBTools bioinformatics toolkit^14^ in order to align three reference genomes simultaneously. Reference genomes used for *Arabidopsis* were the Araport 11 assembly for Arabidopsis (TAIR), the Joint Genomics Institute genome assembly for *R. irregularis* (Genbank: GCA_000439145.3), and a synthetic reference genome containing the T-DNA sequence as well as the *mCherry* selection marker and *AtUBQ10* and *2X35S* promoter sequences. *Lotus* assembly used the Gifu V1.2 assembly (GCA_012489685.2) and a synthetic genome containing the *CYCLOPS/LjIPD3* sequence. Reads were assigned to one of three reference genomes based on the best alignment score and ambiguous reads were discarded. Reads that aligned to an annotated feature in the genome were summarized using featureCounts^15^. Genes with zero counts or with overall low abundance in all samples (<10 read counts) were subsequently filtered prior to downstream analysis.

Gene expression networks were constructed using WGCNA v1.69^16,17^ using log-transformed reads in counts per million (CPM) obtained in R via the cpm function edgeR v3.35.0^18, 19, 20^. Genes with fewer than two read counts for four or more samples were removed from the datasets to reduce spurious correlations. The Pearson correlation metric was used to calculate expression similarity before a signed adjacency matrix was constructed with a soft-threshold power of 16 for *Arabidopsis* samples and 24 for *Lotus*. The topological overlap and topological overlap dissimilarity matrix were calculated from each species’ network adjacency matrix and used to perform average linkage hierarchical clustering with a dynamic tree cutting algorithm to generate modules. Correlation coefficients were calculated between the eigengenes of each module and relevant growth conditions (*i*.*e*., nutrient level) or gene expression to identify module-trait relationships.

Gene Ontology enrichment was performed with PANTHER^21^. GO reduction to produce figure 4B used the following sequence: heirarchical clusters for GO terms in each module were generated in PANTHER and the most-specific term in each cluster was collected. The top 3 most-significant (FDR-adjusted p-value) and most-enriched (fold enrichment) terms in each module were collected and combined, then subjected to semantic similarity clustering in reviGO with a cutoff of 0.5 to produce a list of 25 representative terms^22^.

Differential expression analysis was performed in R using the edgeR package^15, 20^. The estimateGLMCommonDisp function was used to test for differential expression among pairwise treatment groups and significance was evaluated based on the Benjamini-Hochberg FDR adjusted p-value <0.0. Cross-comparison of gene lists from differential expression or network analysis was executed with the VLOOKUP formula in Excel. Figures 3 and 4B were generated in R; all other plots and figures were created in Excel and Powerpoint.

References:

1: [Yano, K., Yoshida, S., Müller, J., Singh, S., Banba, M., Vickers, K., Markmann, K., White, C., Schuller, B., Sato, S., et al. (2008). CYCLOPS, a mediator of symbiotic intracellular accommodation. *Proc. Natl. Acad. Sci.* 105:20540–20545.](https://www.zotero.org/google-docs/?iPmXrX)

2: Dalal, J., Yalamanchili, R., La Hovary, C., Ji, M., Rodriguez-Welsh, M., Aslett, D., Ganapathy, S., Grunden, A., Sederoff, H., and Qu, R. (2015). A novel gateway-compatible binary vector series (PC-GW) for flexible cloning of multiple genes for genetic transformation of plants. *Plasmid* 81:55–62.

3: [Ivanov, S., and Harrison, M. J. (2014). A set of fluorescent protein-based markers expressed from constitutive and arbuscular mycorrhiza-inducible promoters to label organelles, membranes and cytoskeletal elements in *Medicago truncatula*. *Plant J.* 80:1151–1163.](https://www.zotero.org/google-docs/?iPmXrX)

4: Nagaya, S., Kawamura, K., Shinmyo, A., and Kato, K. (2010). The *HSP* terminator of *Arabidopsis thaliana* increases gene esxpression in plant cells. *Plant & Cell Physiology* 51: 328-332.

5: [Singh, S., Katzer, K., Lambert, J., Cerri, M., and Parniske, M. (2014). CYCLOPS, A DNA-Binding Transcriptional Activator, Orchestrates Symbiotic Root Nodule Development. *Cell Host Microbe* 15:139–152.](https://www.zotero.org/google-docs/?iPmXrX)

6: Davis, A. M., Hall, A., Millar, A. J., Darrah, C., and Davis, S. J. (2009). Protocol: Streamlined sub-protocols for floral-dip transformation and selection of transformants in Arabidopsis thaliana. *Plant Methods* 5:3.

7: Porebski, S., Bailey, L.G., and Baum, B.R. (1997). Modification of CTAB DNA extraction protocol for plants containing high polysaccharide and polyphenol components. *Plant Molecular Biology Reporter* 15:8-15.

8: Wisniewski J. R., Zougman A., Nagaraj N. and Mann M. (2009) Universal sample preparation method for proteome analysis. *Nat. Comm.* 6(5) 359-362.

9: Shevchenko A., Tomas H., Havli J., Olsen J.V. and Mann M. (2006) In-gel digestion for mass spectrometric characterization of proteins and proteomes. *Nature Protocols* 1(6) 2856- 2860.

10: Mordant A. and Kleiner M. (2021) Evaluation of sample preservation and storage methods for
 metaproteomics and analysis of intestinal microbes *Microbiol Spectrum* 9(3) 01877-21

11: Zybailov B, Mosley AL, Sardiu ME, Coleman MK, Florens L, Washburn MP. 2006.
 Statistical analysis of membrane proteome expression changes in *Saccharomyces
 cerevisiae*. *J Proteome Res* 5:2339–2347
12: Chen, Y., Chen, Y., Shi, C., Huang, Z., Zhang, Y., Li, S., Li, Y., Ye, J., Yu, C., Li, Z., et al. (2018). SOAPnuke: a MapReduce acceleration-supported software for integrated quality control and preprocessing of high-throughput sequencing data. *GigaScience* 7.
13: Andrews, S. (2010). FastQC:  A Quality Control Tool for High Throughput Sequence Data Advance Access published 2010.

14: Bushnell, B. 2014. “BBMap: A Fast, Accurate, Splice-Aware Aligner.”

15: Liao, Y., Smyth, G. K., and Shi, W. (2014). featureCounts: an efficient general purpose program for assigning sequence reads to genomic features. *Bioinformatics* 30:923–930.

16: Langfelder, P., and Horvath, S. (2008). WGCNA: an R package for weighted correlation network analysis. *BMC Bioinformatics* 9:559.

17: Langfelder, P., and Horvath, S. (2012). Fast *R* Functions for Robust Correlations and Hierarchical Clustering. *J. Stat. Soft.* 46.

18: Chen, Y., Lun, A. T. L., and Smyth, G. K. (2016). From reads to genes to pathways: differential expression analysis of RNA-Seq experiments using Rsubread and the edgeR quasi-likelihood pipeline. *F1000Res* 5:1438.

19: McCarthy, D. J., Chen, Y., and Smyth, G. K. (2012). Differential expression analysis of multifactor RNA-Seq experiments with respect to biological variation. *Nucleic Acids Research* 40:4288–4297.

20: Robinson, M. D., McCarthy, D. J., and Smyth, G. K. (2010). edgeR: a Bioconductor package for differential expression analysis of digital gene expression data. *Bioinformatics* 26:139–140.

21: Mi, H., Muruganujan, A., Casagrande, J. T., and Thomas, P. D. (2013). Large-scale gene
function analysis with the PANTHER classification system. *Nat Protoc* 8:1551–1566.

22: Supek, F., Bošnjak, M., Škunca, N., and Šmuc, T. (2011). REVIGO Summarizes and
Visualizes Long Lists of Gene Ontology Terms. *PLoS ONE* 6:e21800.
