# Supplementary figures and images for: IPD3, a master regulator of arbuscular mycorrhizal symbiosis, affects genes for immunity and metabolism of non-host Arabidopsis when restored long after its evolutionary loss
Source: Plant Mol Biol. 2024 Feb 18;114(2):21. doi: 10.1007/s11103-024-01422-3 (PMC10874911; doi:10.1007/s11103-024-01422-3)

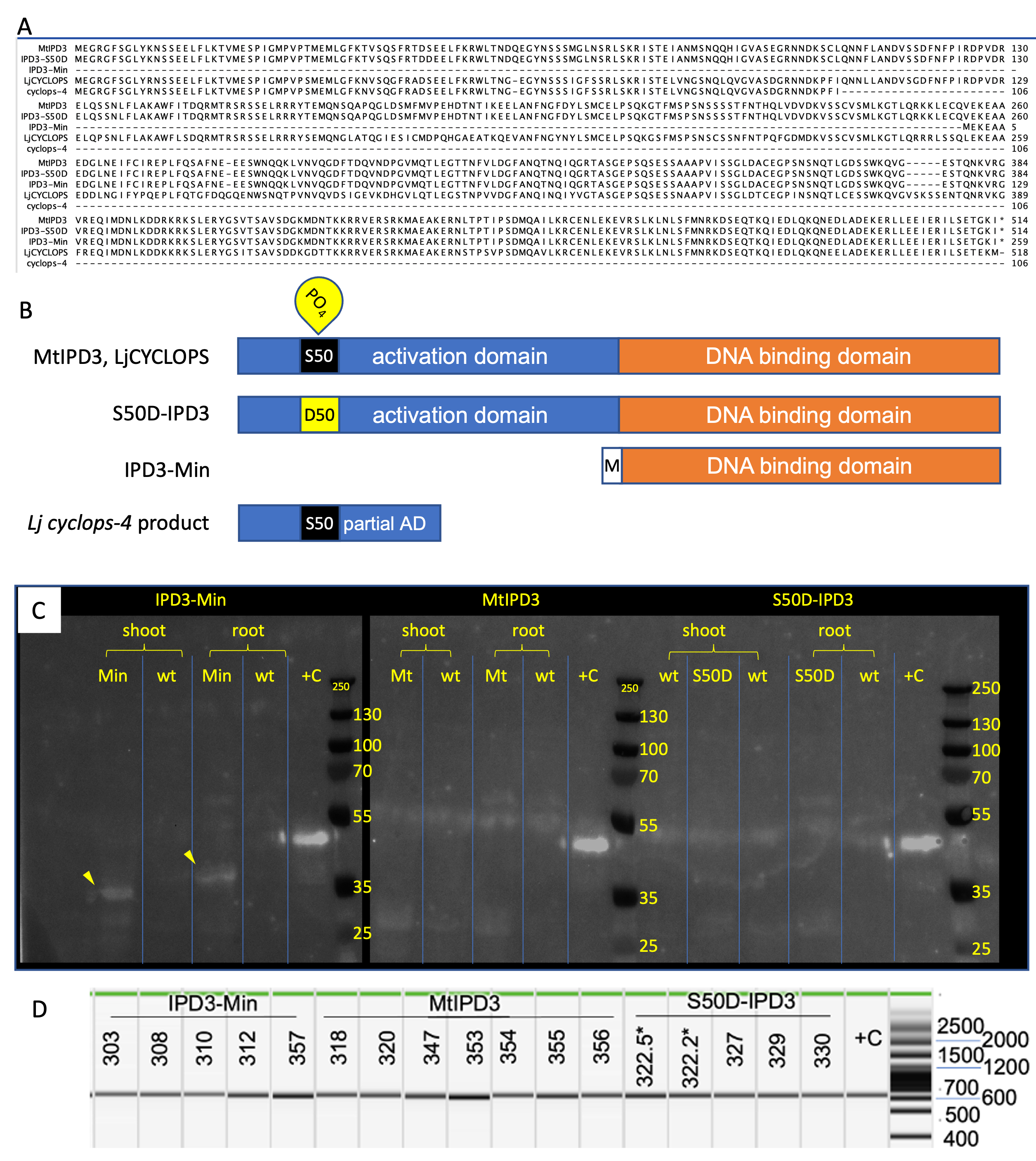

Supplement: Supplementary file 2 — Supplementary file2 (PNG 3327 KB) Online Resource 2 Comparison of IPD3 protein versions used in this study and validation of RNA and protein expression in transgenic plants. The +C control sample is a synthetic peptide of the 349 C-terminal residues of MtIPD3 with expected size 39.98 kDa; expected size of IPD3-Min is 29.19 kDa and expected size of full-length IPD3 versions is 57.98 kDa. (A) Protein sequence alignment of Medicago truncatula IPD3, S50D phosphomimic IPD3, constitutively active DNA binding domain IPD3Min, Lotus japonicus IPD3/CYCLOPS, and the truncated C-terminal fragment resulting from the cyclops-4 mutation in Lotus. (B) Schematic of protein domains as affected by variants in this study. (C) Western blot of 3 IPD3 versions from transgenic Arabidopsis roots and shoots. This blot was visualized with fluorescent secondary antibody under 488/530 nm excitation/emission conditions (see Methods and Online Resource 1), causing the target protein bands to appear as light areas of high exposure while protein size ladder bands are visualized by dye and appear dark.(D) rt-PCR to confirm expression of IPD3 versions as RNA in leaves of T3 individuals. [file 11103_2024_1422_MOESM2_ESM.png]

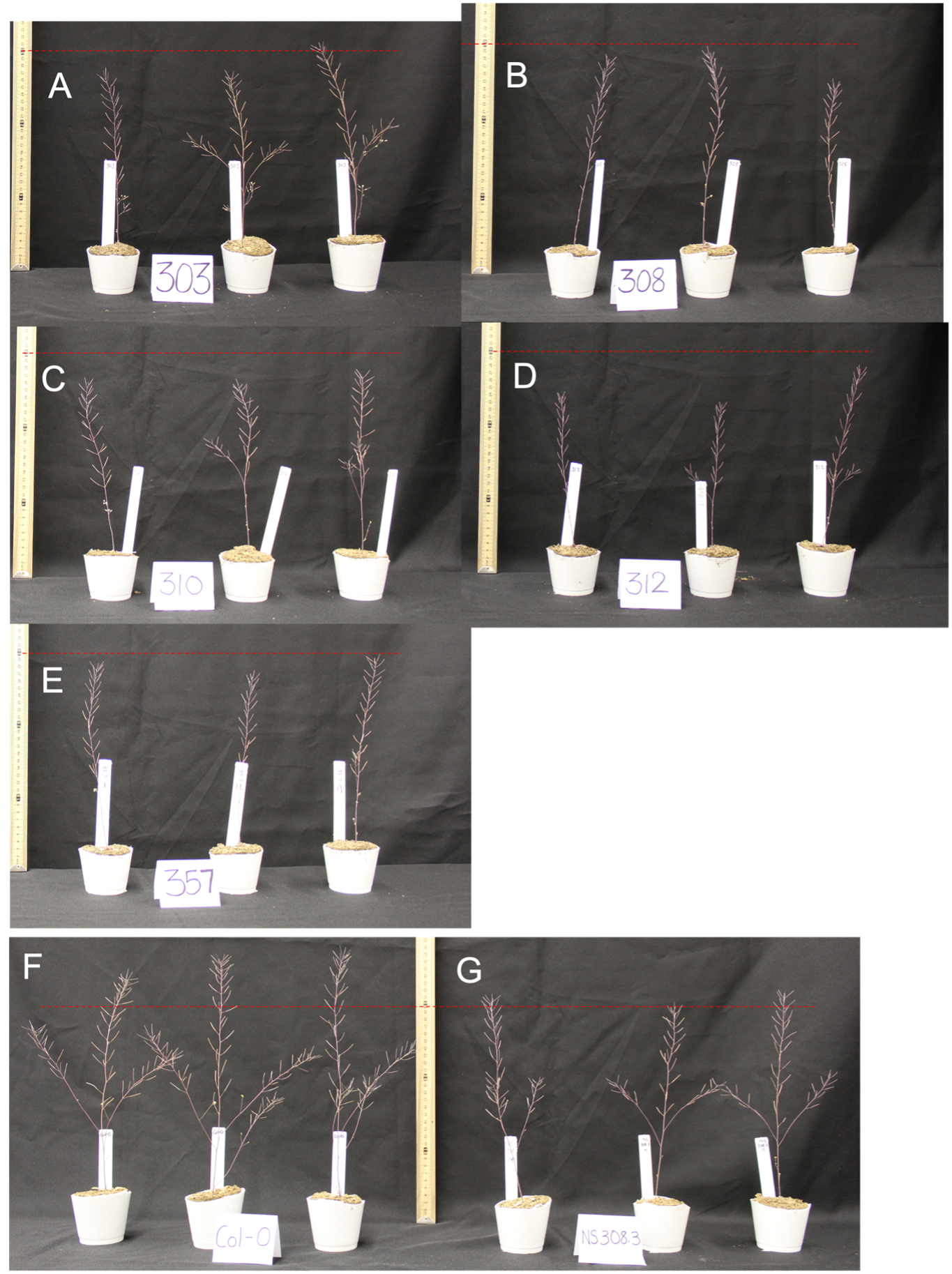

Supplement: Supplementary file 4 — Supplementary file4 (PNG 3488 KB) Online Resource 4 Mature phenotypes of IPD3-Min transgenic and control Arabidopsis lines in T3. (A-E) Transgenic lines 303, 308, 310, 312, 357, respectively; (F) wild type Col-0; (G) null segregants of transgenic line 308. Red line in all pictures shows 30 cm from surface [file 11103_2024_1422_MOESM4_ESM.png]

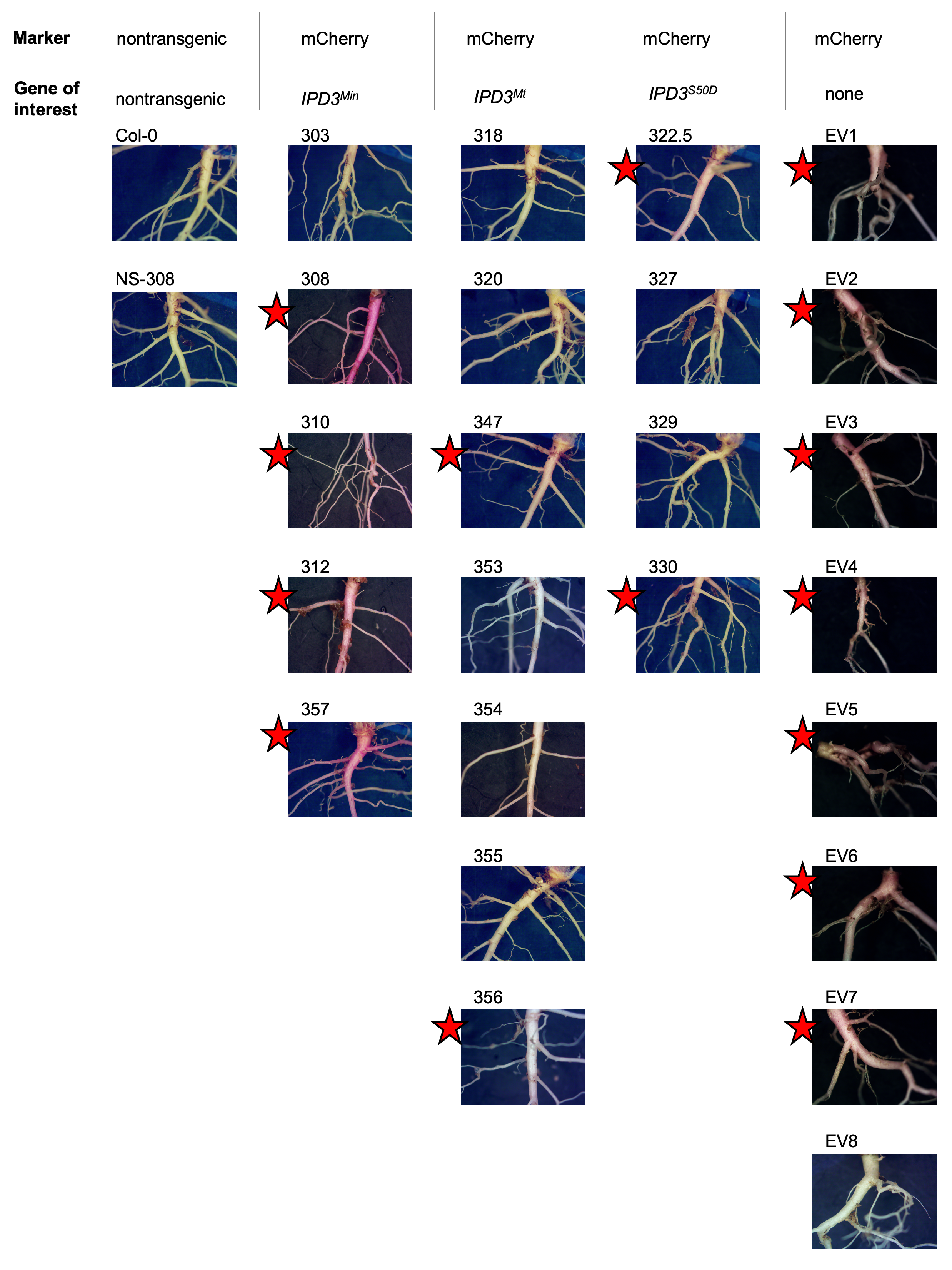

Supplement: Supplementary file 6 — Supplementary file6 (PNG 5009 KB) Online Resource 6 Representative root images of transgenic and control lines. In an original set of experiments, the roots of soil-grown IPD3Min transgenic plants were bright pink while wild type and null segregant controls were white, and IPD3Mt and IPD3S50D were faint pink to white. In a followup experiment, multiple independent empty vector controls containing the mCherry marker also ranged from pink to white. Red stars mark those root images visually assessed to show any degree of pink coloration. [file 11103_2024_1422_MOESM6_ESM.png]

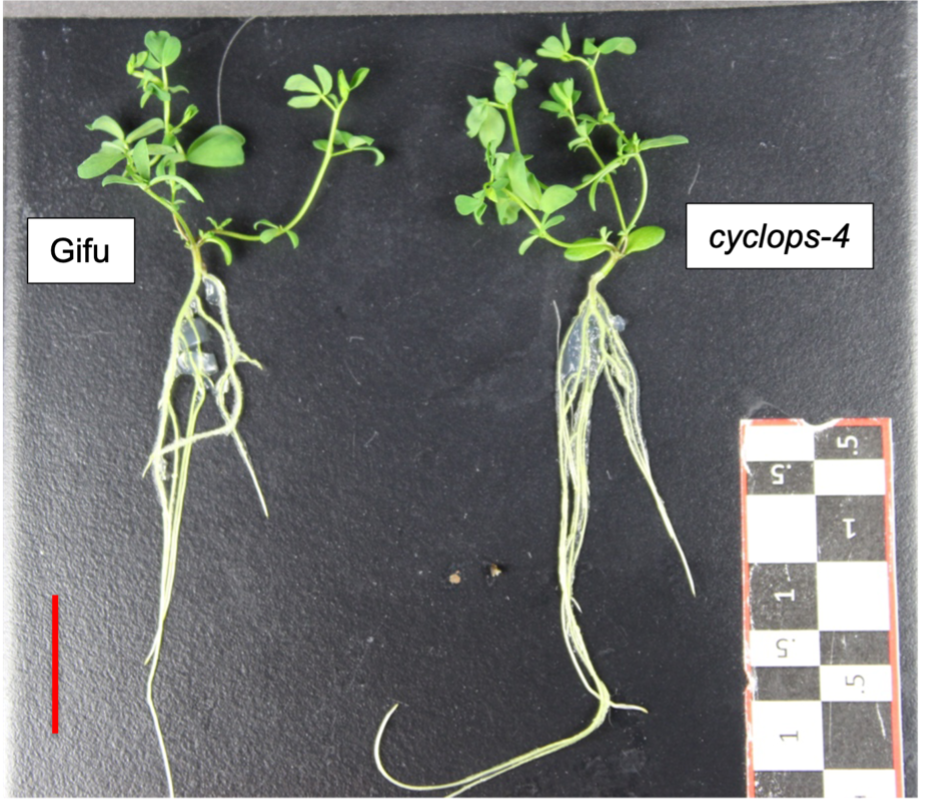

Supplement: Supplementary file 7 — Supplementary file7 (PNG 1262 KB) Online Resource 7 Images of 5-week-old Lotus japonicus seedlings of Gifu wildtype and cyclops-4 knockout mutant of ipd3 as grown on sterile petri dishes for the transcriptome experiment. Scale bar = 2 cm (scale tiles within image are denoted in cm) [file 11103_2024_1422_MOESM7_ESM.png]

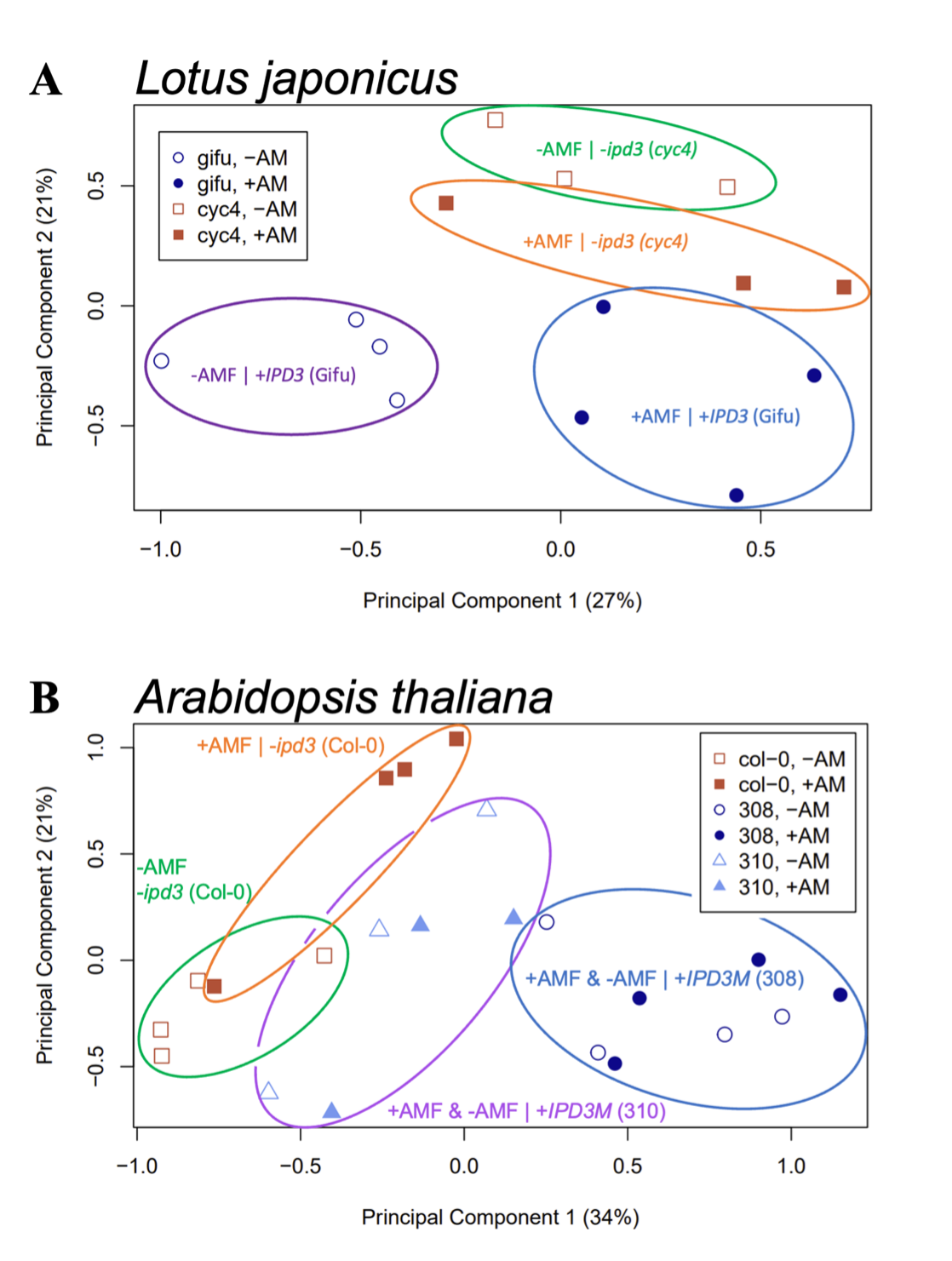

Supplement: Supplementary file 8 — Supplementary file8 (PNG 455 KB) Online Resource 8 Principal component analysis shows clustering of transcriptomes by IPD3 genotype and AMF treatment in two species. (A) Lotus Gifu samples cluster according to AMF treatment along PC1. Gifu and cyclops-4 samples cluster by genotype along PC2. In contrast to Gifu, cyclops-4 plants do not separate along PC1 according to AMF treatment; both treatment groups instead cluster near AMF-treated Gifu plants along this axis. (B) Arabidopsis samples cluster by genotype along PC1 (IPD3M = IPD3Min transgenic). While wild type Col-0 plants cluster by AMF treatment along PC2, there is no equivalent separation by AMF treatment within either IPD3Min line (308; 310) [file 11103_2024_1422_MOESM8_ESM.png]

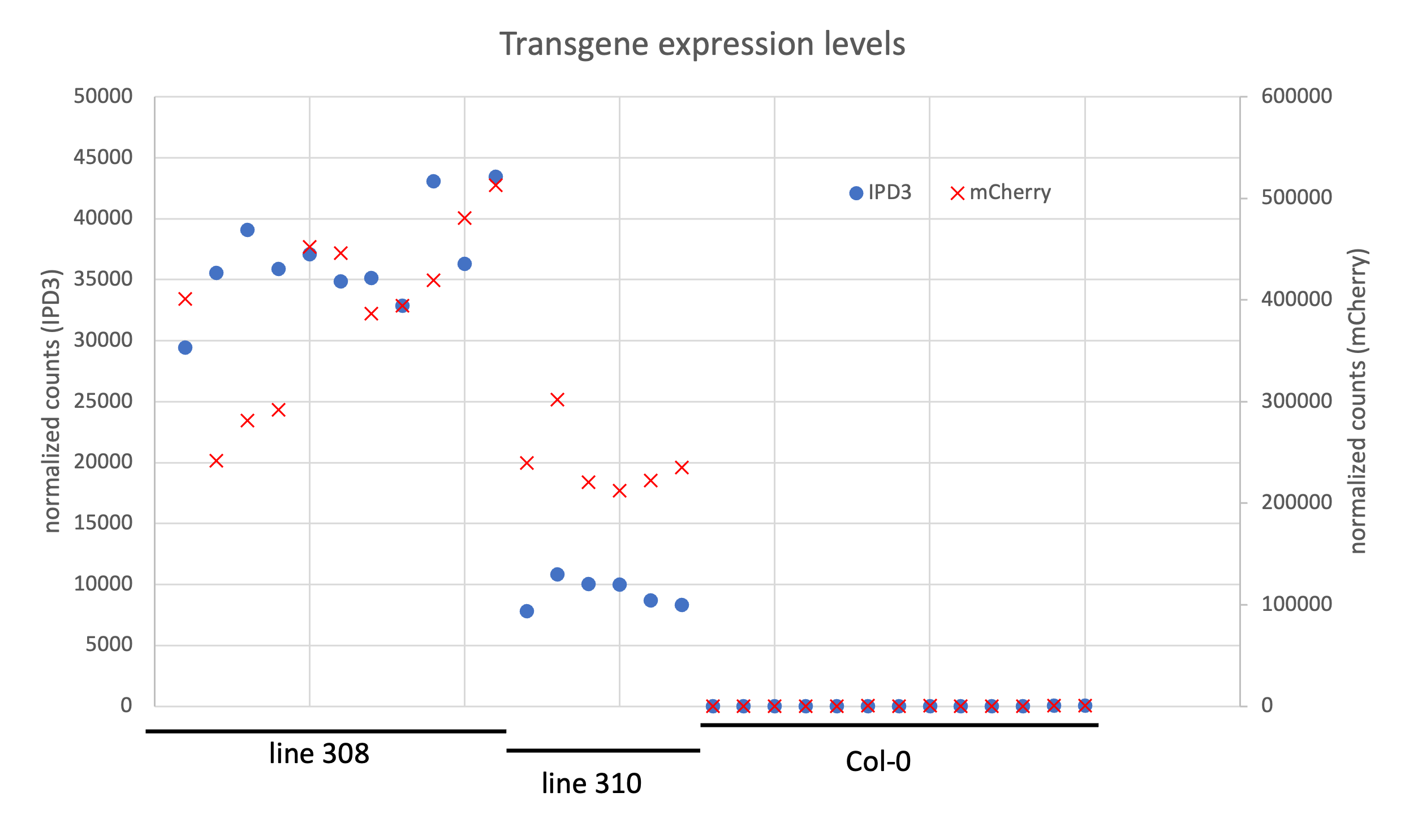

Supplement: Supplementary file 9 — Supplementary file9 (PNG 172 KB) Online Resource 9 IPD3-Min and mCherry transcript level in Arabidopsis transcriptome samples. Average IPD3-Min transcript count in line 308 is 36,591, ~4X higher than line 310 with average IPD3-Min count of 9,250 [file 11103_2024_1422_MOESM9_ESM.png]

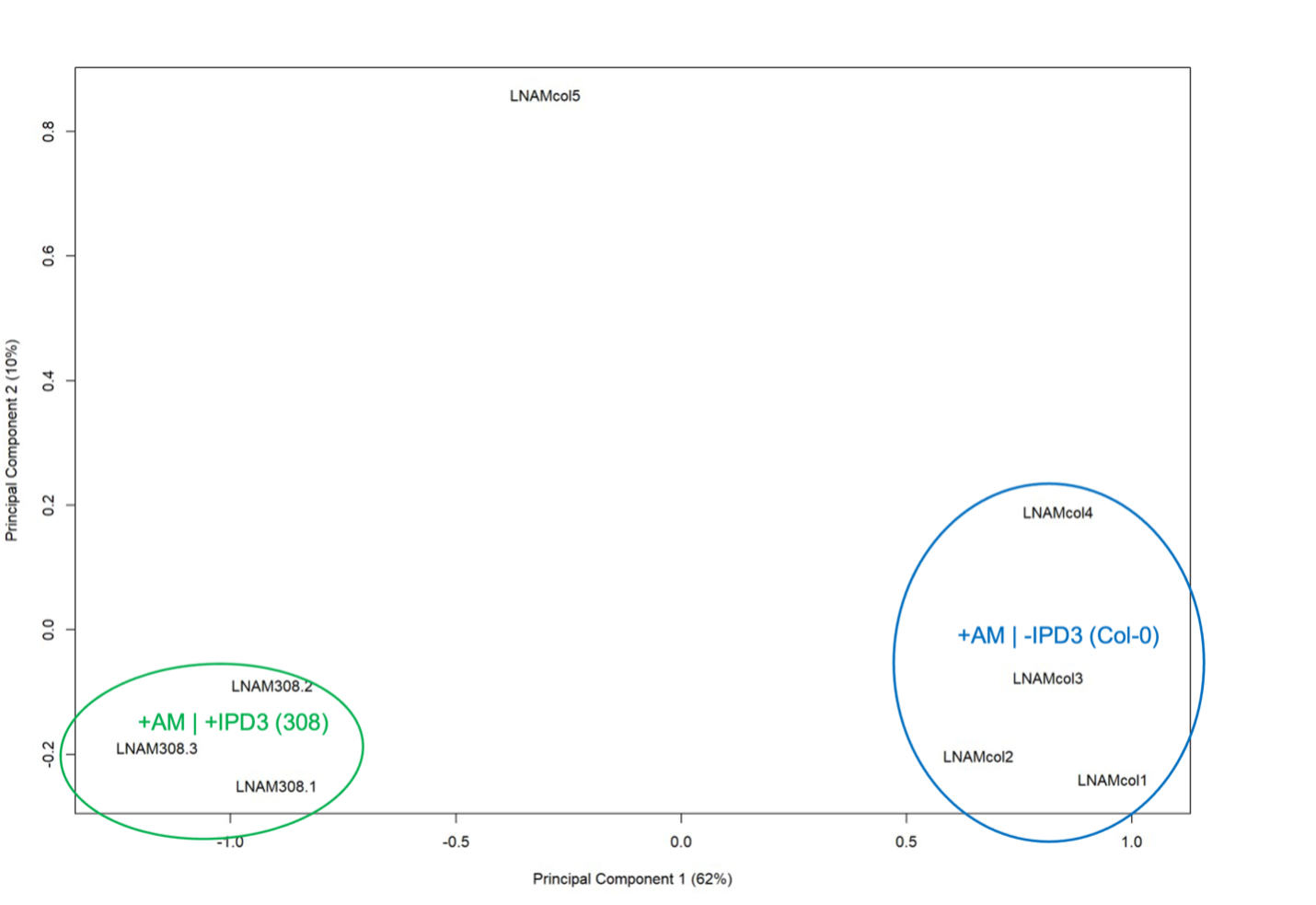

Supplement: Supplementary file 10 — Supplementary file10 (PNG 181 KB) Online Resource 10 Principal Component Analysis of Arabidopsis transcriptomes under low-nutrient conditions with AMF treatment. Only IPD3Min transgenic line 308 was used for the low-nutrient experiment. Transcriptomes cluster by IPD3 genotype along PC1, which accounts for 62% of variation [file 11103_2024_1422_MOESM10_ESM.png]

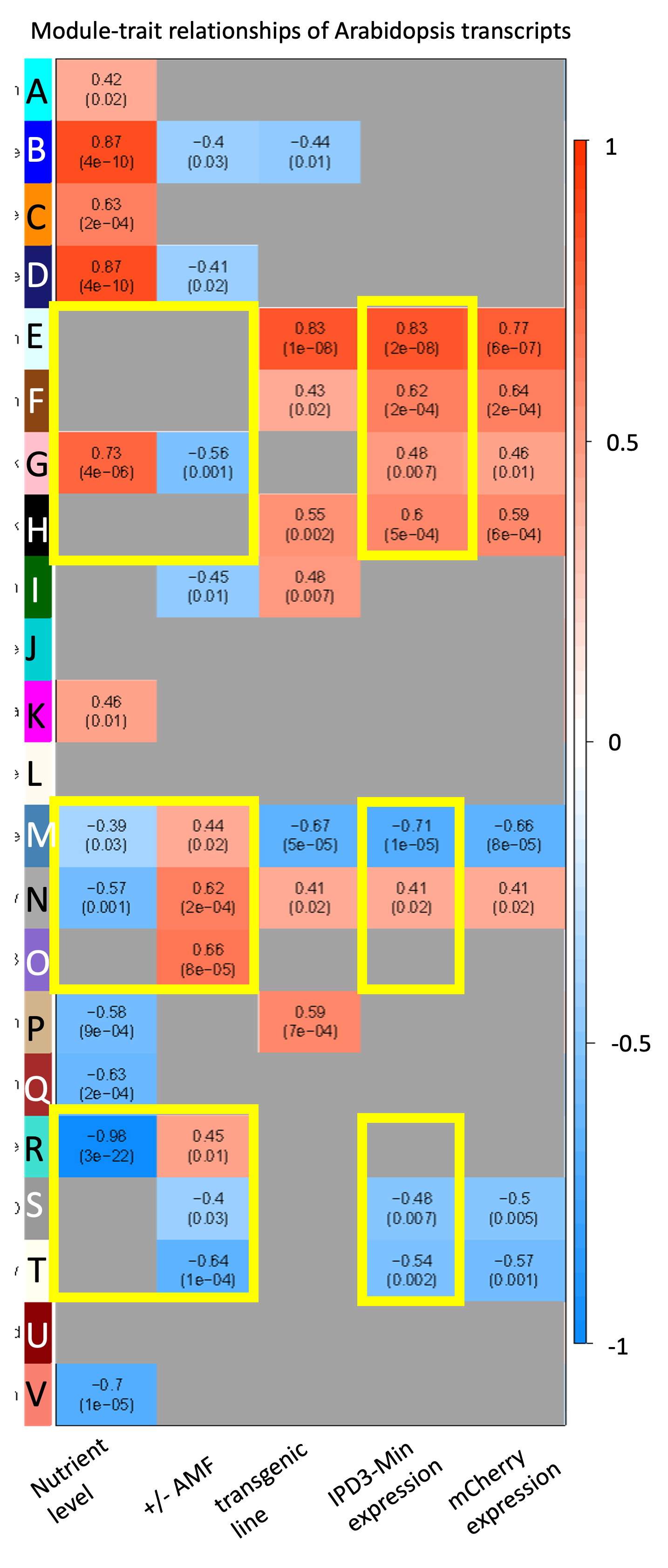

Supplement: Supplementary file 11 — Supplementary file11 (PNG 672 KB) Online Resource 11 Heatmap of complete gene expression correlation network (WGCNA) with module-trait correlations. Cells included in figure 3 of the main text are highlighted in yellow. The top number in each cell is the signed eigengene correlation of that module to value of trait shown on the X axis. A value of 1 indicates perfect positive correlation, -1 would indicate perfect inverse correlation. Bottom value shown in parentheses is the P-value of that module-trait correlation. Trait/treatment labels are as follows: Nutrient Level: determined by medium composition as described in Methods, positive correlation of a module to Nutrient Level indicates positive correlation of gene expression for module members with combined macronutrient concentration; +/-AMF: determined by presence or absence of germinated AM fungus spore treatment, positive correlation of a module to +/-AMF indicates positive correlation of gene expression for module members with presence of AMF; Transgenic Line: determined by transgenic genotype, discriminating between independent IPD3Min lines 308 and 310. Positive or negative correlation to Transgenic Line indicates that gene expression for that module is affected by transgenic genotype but is non-quantitative (see following); IPD3-Min Expression: determined by quantitative transcript level of IPD3Min, agnostic of line identity. Positive correlation of a module with IPD3-Min Expression indicates positive correlation of gene expression for module members with level of IPD3Min expression across all samples. Correlation of a module to Transgenic Line but not IPD3-Min Expression (i.e. Modules B, I, P), or correlation in opposite directions (not observed), would suggest that effects in that module could be due to insertion site effects rather than IPD3Min expression itself; mCherry Expression: determined by quantitative transcript level of mCherry marker gene across all samples. [file 11103_2024_1422_MOESM11_ESM.png]

## Module F

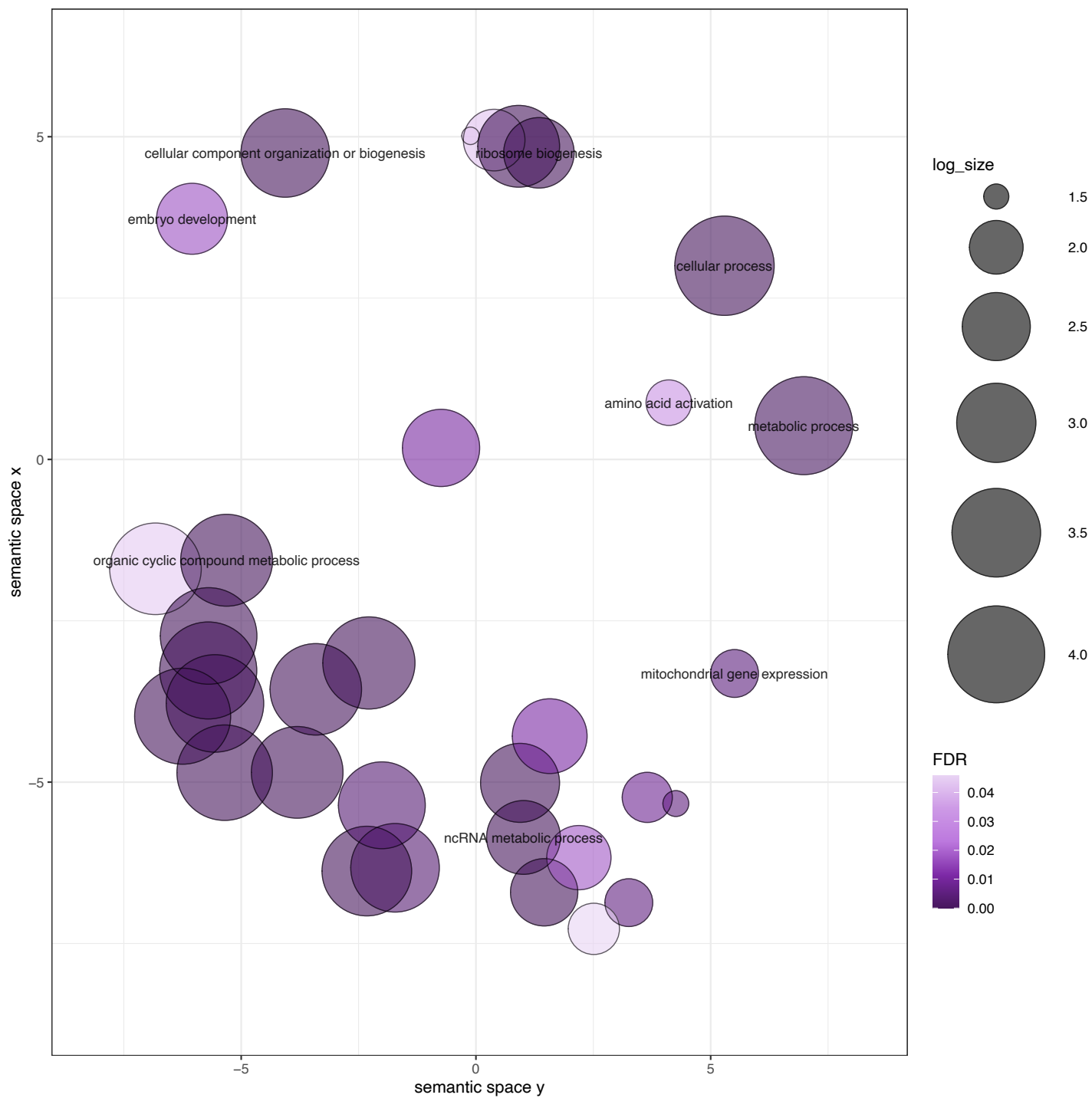

## Module G

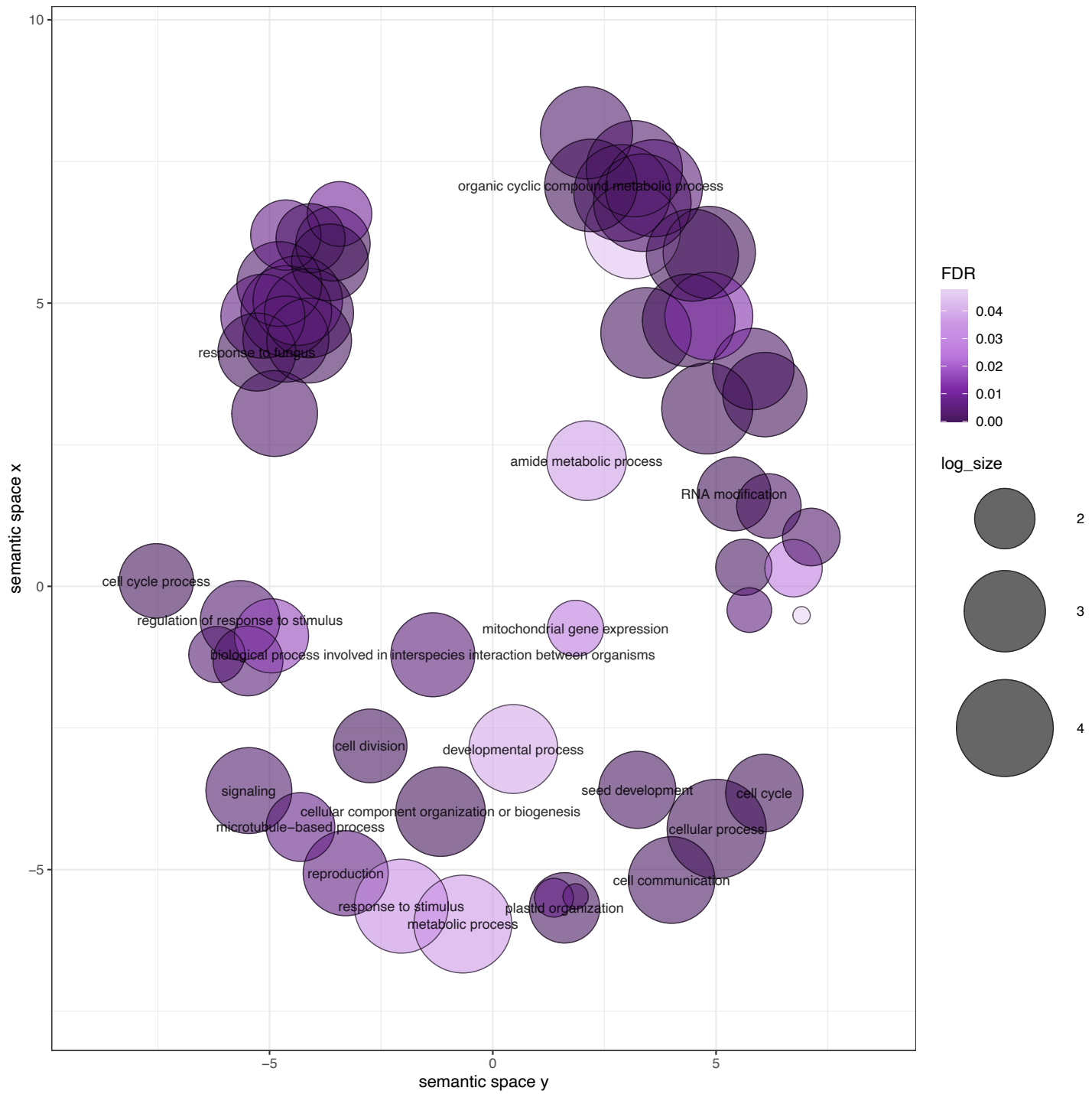

Module H

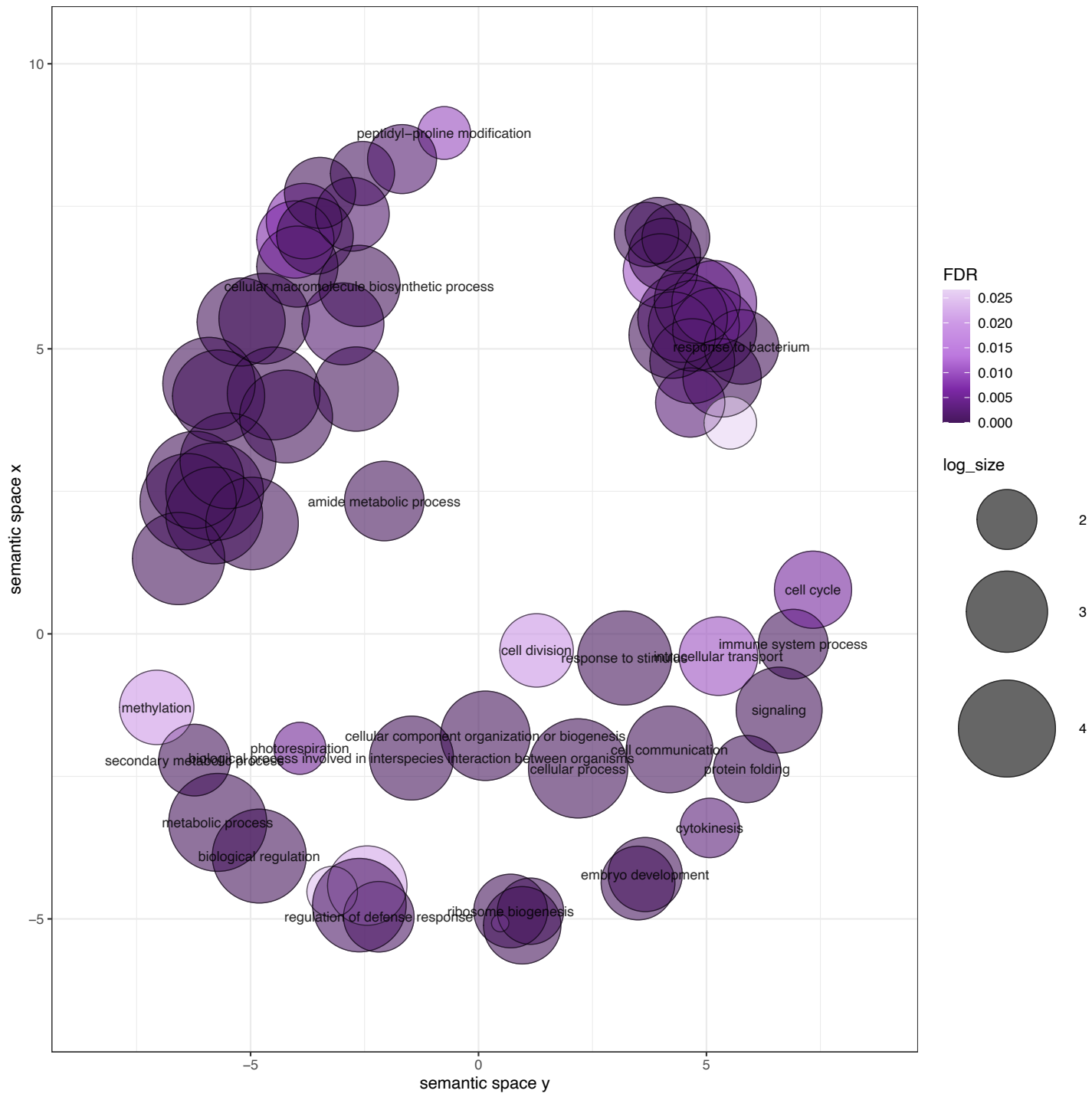

Module M

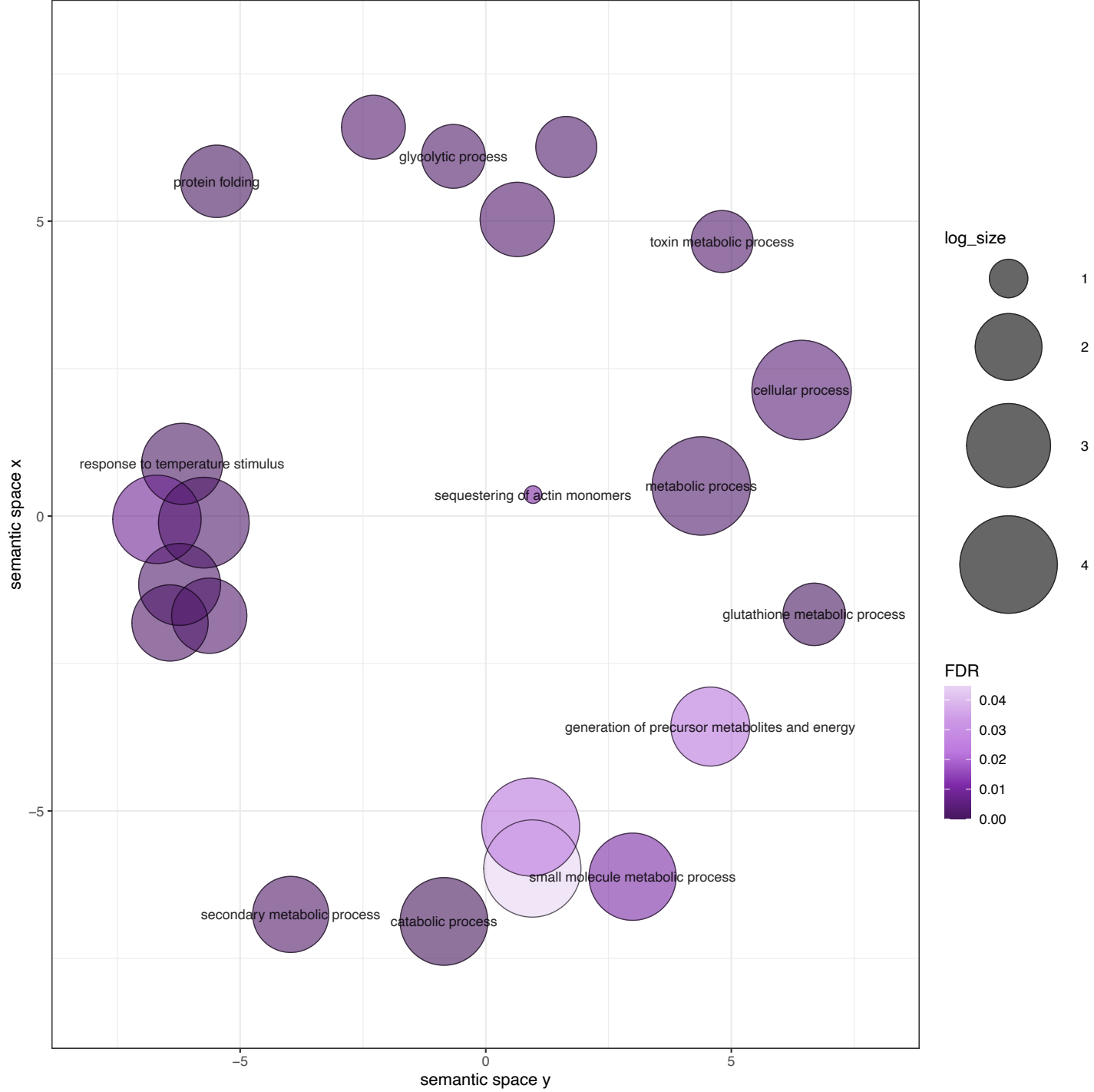

Module N

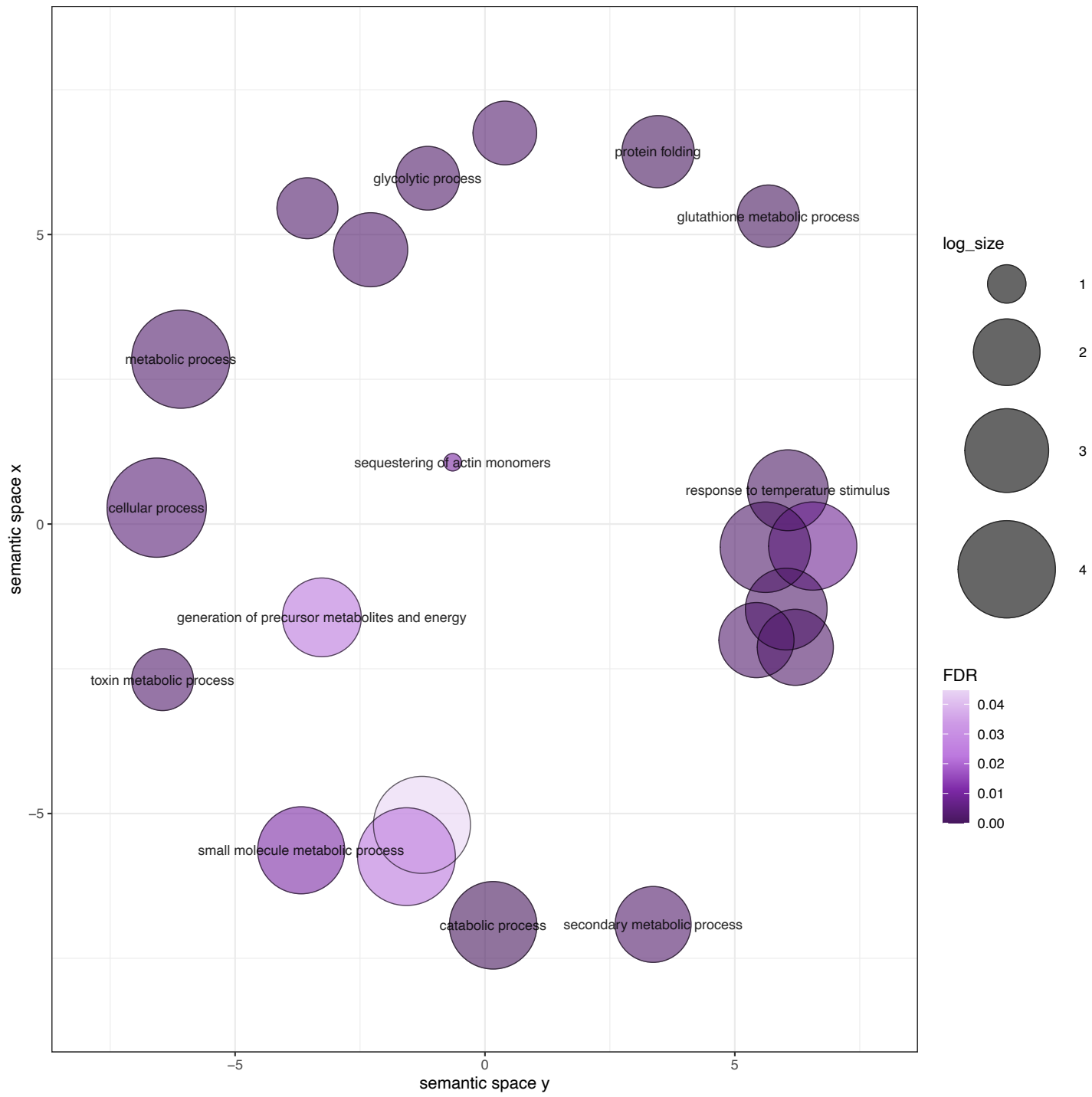

Module O

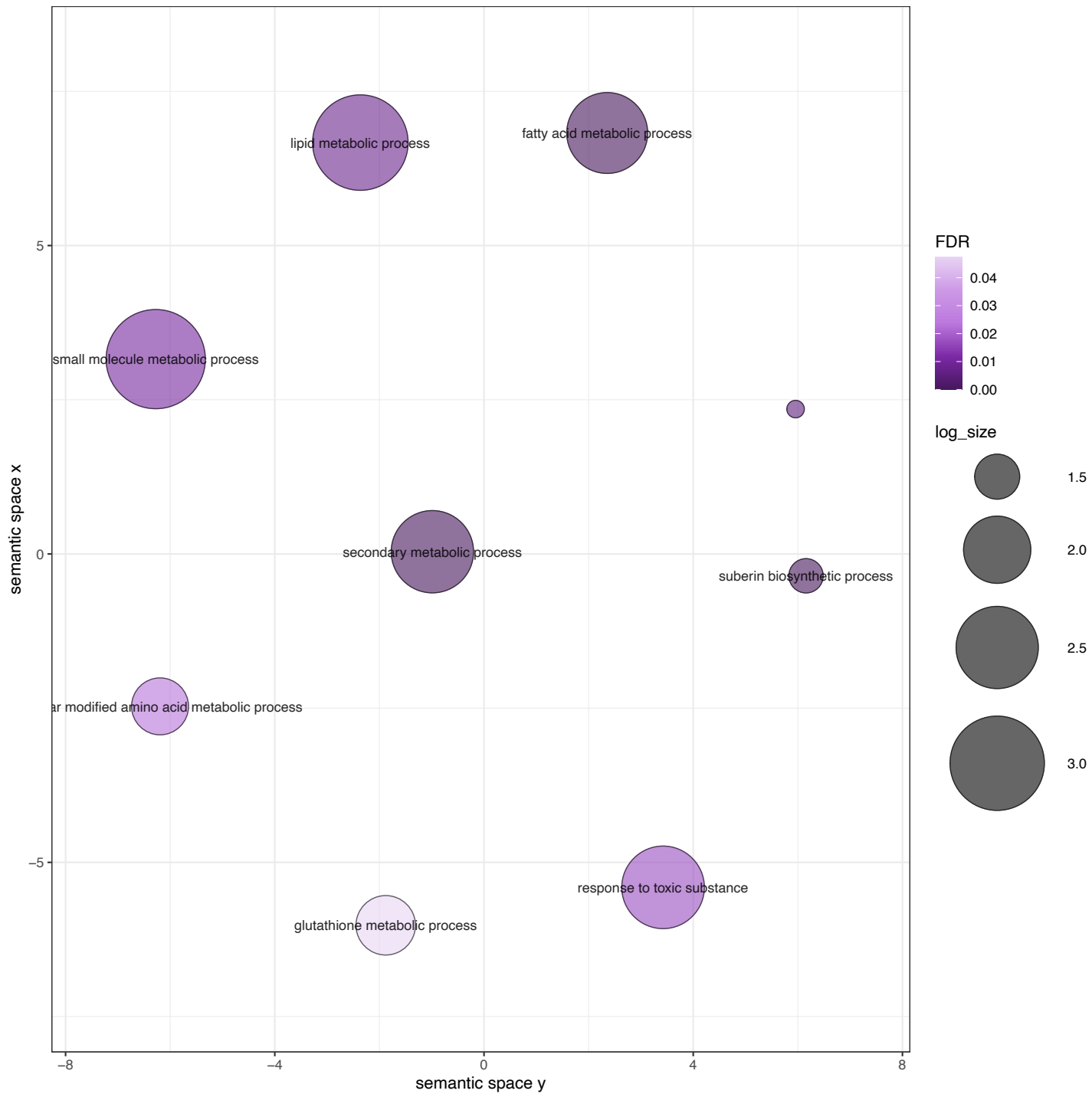

Module R

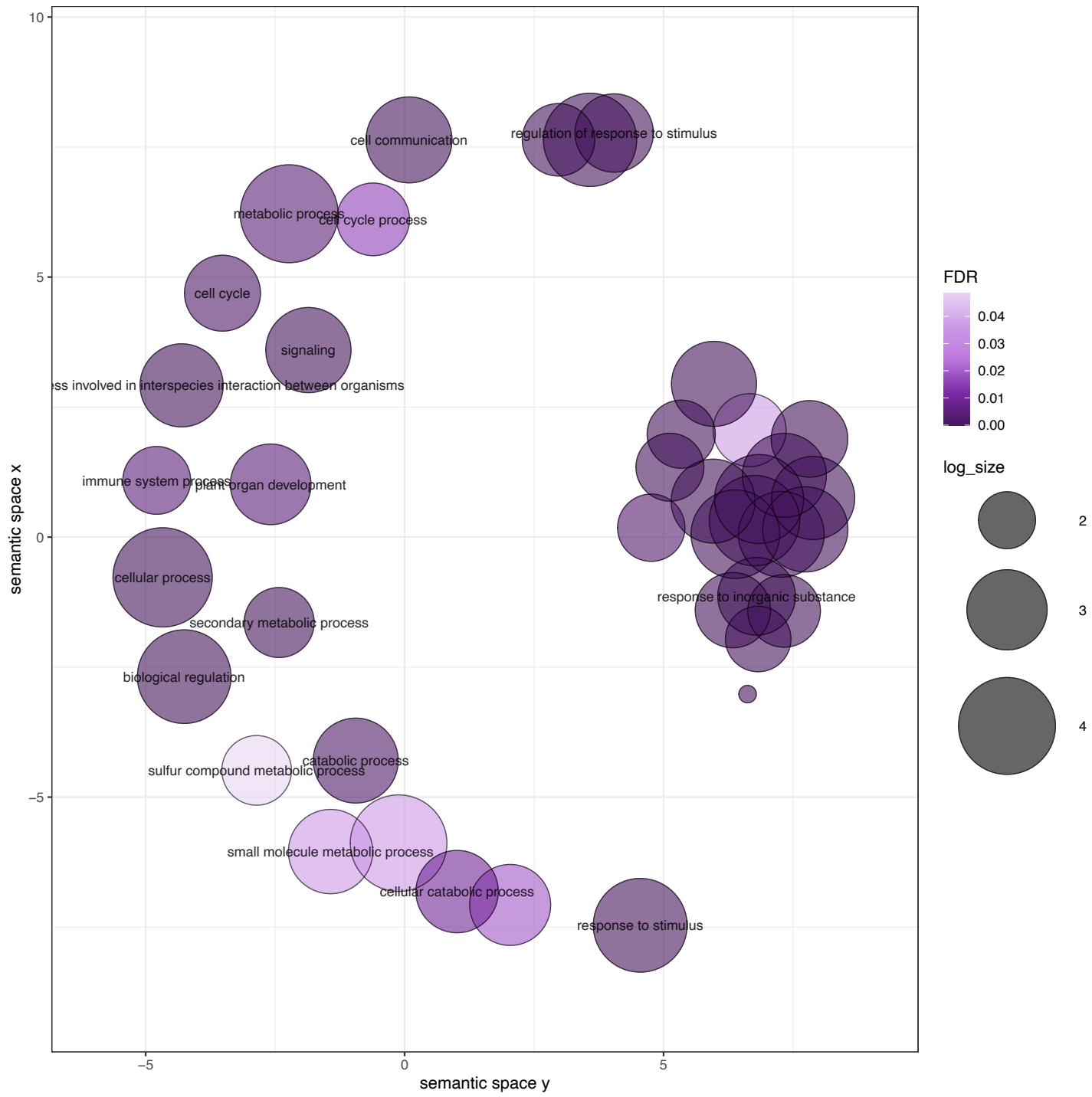

Module S

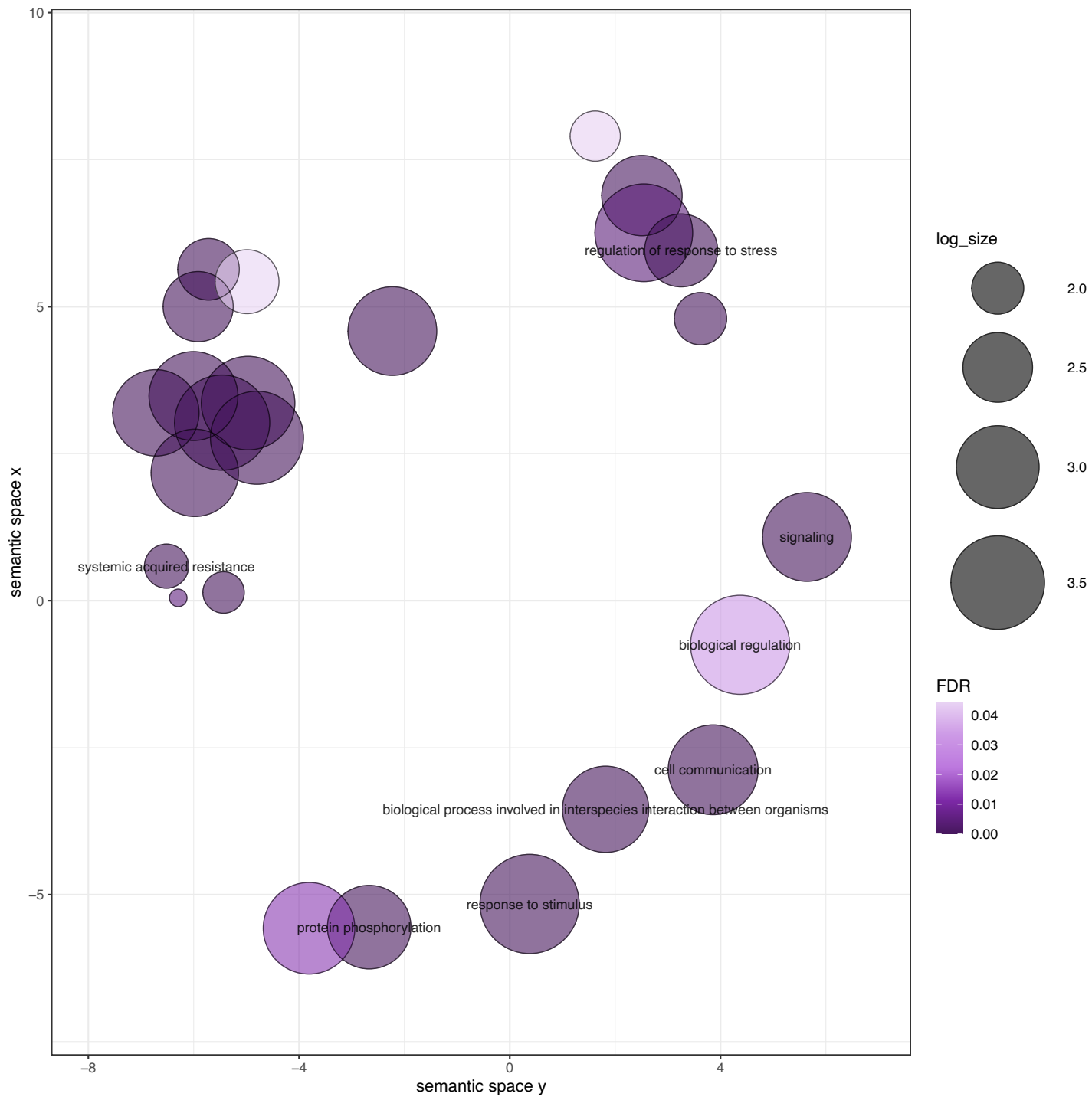

Module T

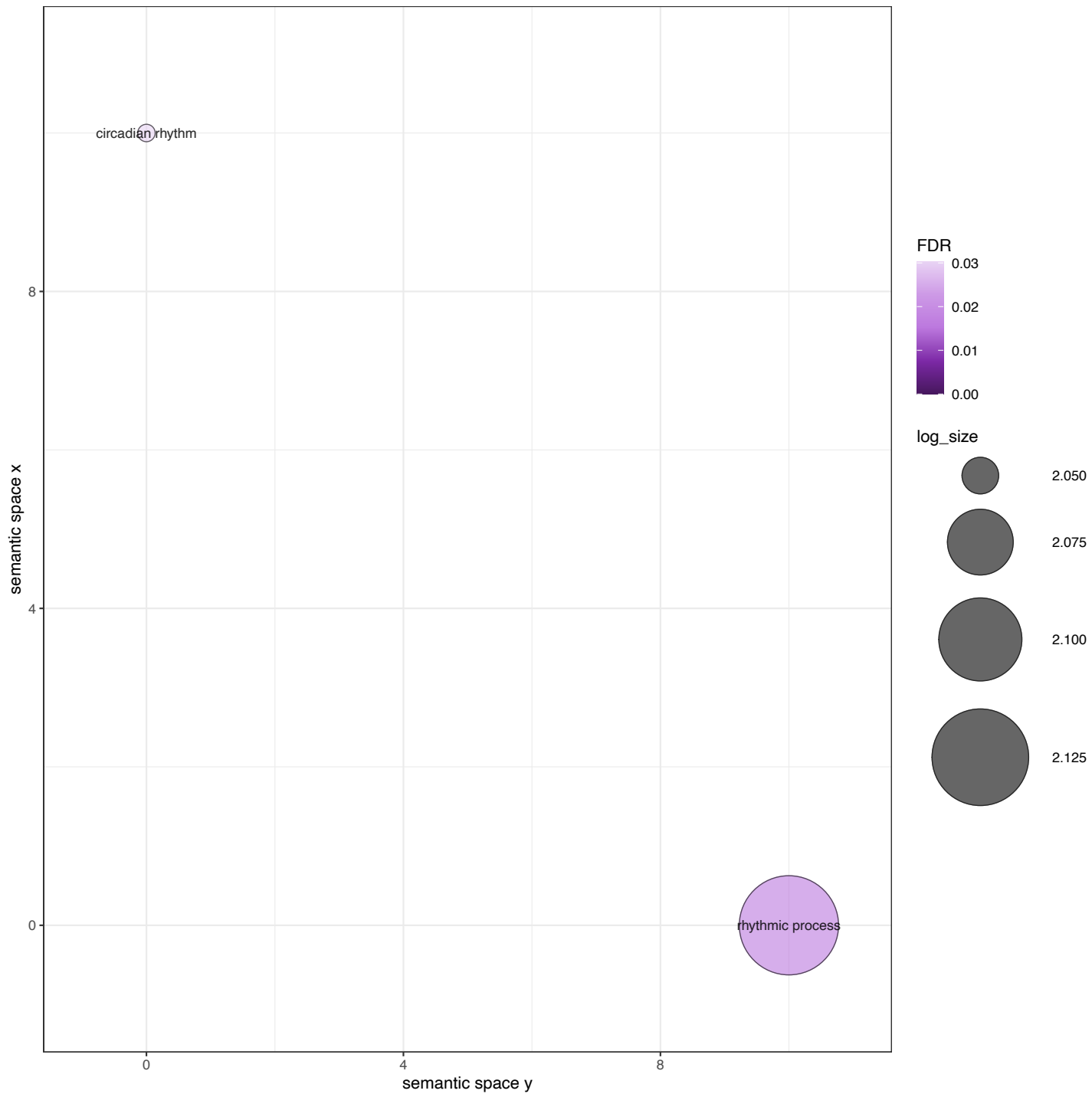

Supplement: Supplementary file 13 — Supplementary file13 (PDF 156 KB) Online Resource 13 Semantic similarity clustering of gene ontology (GO) terms of all modules shown in figure 3 of the main text (including all Arabidopsis modules significantly correlated to IPD3Min expression). Size of the point on the plot for each GO term corresponds to its fold enrichment in that module, and intensity of color corresponds to FDR-corrected p-value of the enrichment. Proximity of the points represents their semantic similarity as determined by ReviGO (Supek et al 2011) from relatedness and frequency within the Arabidopsis gene ontology reference [file 11103_2024_1422_MOESM13_ESM.pdf]

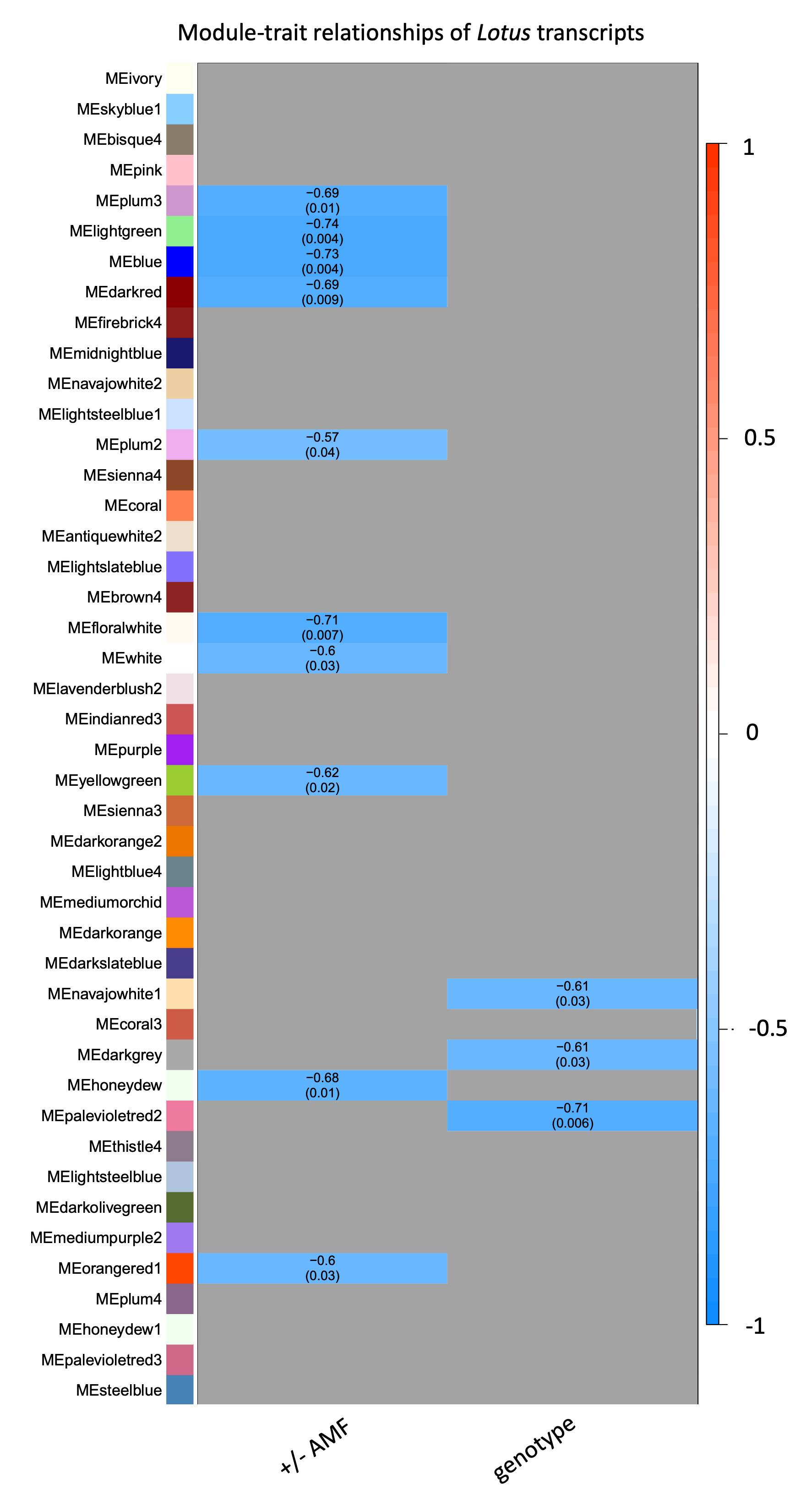

Supplement: Supplementary file 17 — Supplementary file17 (PNG 404 KB) Online Resource 17 Gene co-regulatory modules resulting from WGCNA analysis of Lotus transcriptomes. AMF corresponds to AM fungus treatment. Genotype reflects the presence of functional IPD3 in the wild type with a value of +1 and its absence in the cyclops-4 mutant with a value of 0 [file 11103_2024_1422_MOESM17_ESM.png]

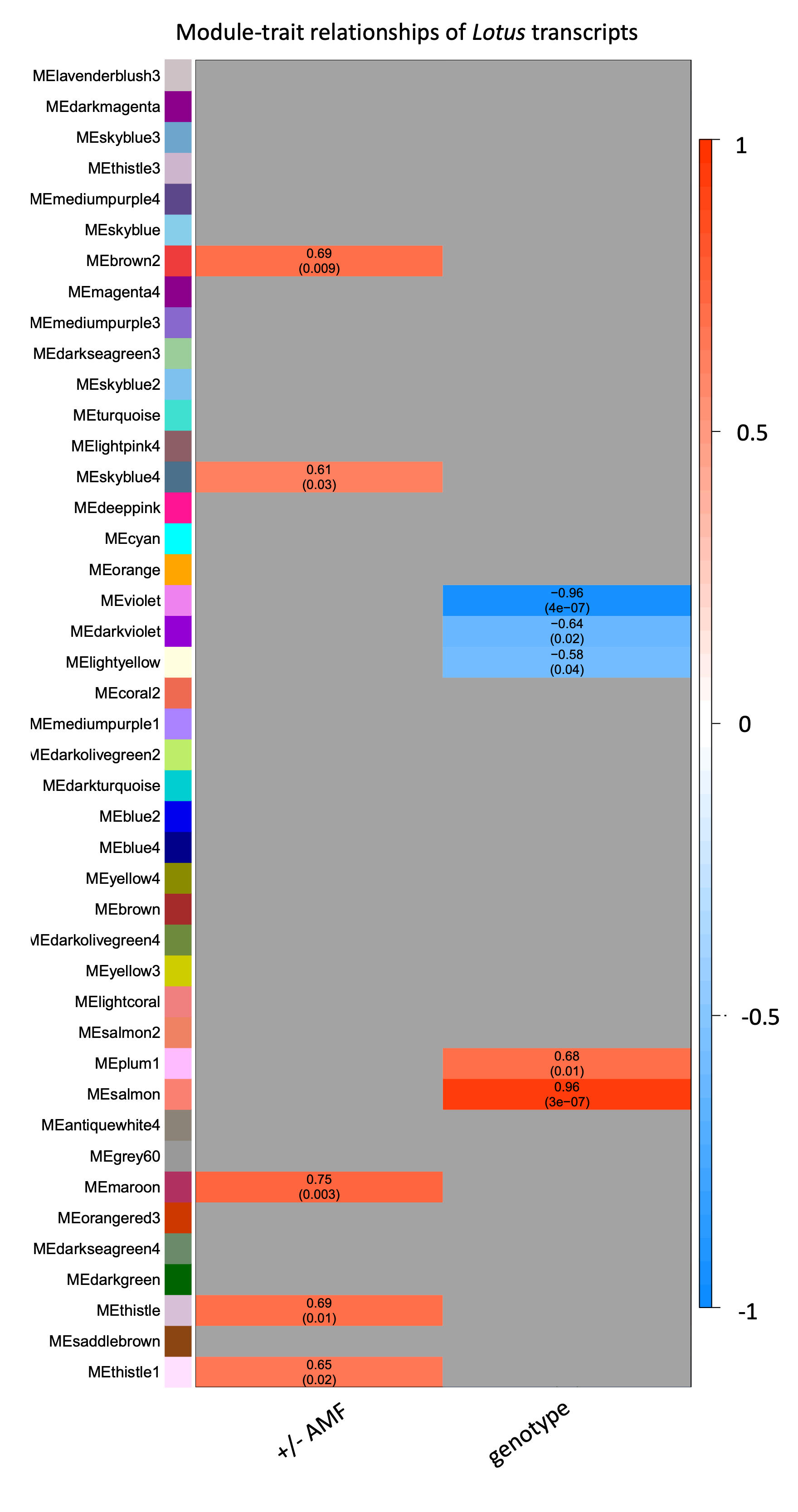

Supplement: Supplementary file 18 — Supplementary file18 (PNG 392 KB) [file 11103_2024_1422_MOESM18_ESM.png]
